# Counting charges on membrane-bound peptides†
†Electronic supplementary information (ESI) available. See DOI: 10.1039/c8sc00804c


**DOI:** 10.1039/c8sc00804c

**Published:** 2018-04-03

**Authors:** Alicia C. McGeachy, Emily R. Caudill, Dongyue Liang, Qiang Cui, Joel A. Pedersen, Franz M. Geiger

**Affiliations:** a Department of Chemistry , Northwestern University , 2145 Sheridan Road , Evanston , IL 60660 , USA . Email: geigerf@chem.northwestern.edu; b Department of Chemistry , University of Wisconsin-Madison , 1101 University Avenue , Madison , WI 53706 , USA; c Environmental Chemistry and Technology Program , University of Wisconsin-Madison , 660 North Park Street , Madison , WI 53706 , USA; d Department of Chemistry , Boston University , 590 Commonwealth Ave. , Boston , MA 02215 , USA; e Department of Soil Science , University of Wisconsin-Madison , 1525 Observatory Drive , Madison , WI 53706 , USA; f Department of Civil & Environmental Engineering , University of Wisconsin-Madison , 1415 Engineering Drive , Madison , WI 53706 , USA

## Abstract

Quantifying the number of charges on peptides bound to interfaces requires reliable estimates of (i) surface coverage and (ii) surface charge, both of which are notoriously difficult parameters to obtain, especially at solid/water interfaces. Here, we report the thermodynamics and electrostatics governing the interactions of l-lysine and l-arginine octamers (Lys_8_ and Arg_8_) with supported lipid bilayers prepared from a 9 : 1 mixture of 1,2-dimyristoyl-*sn-glycero*-3-phosphocholine (DMPC) and 1,2-dimyristoyl-*sn*-glycero-3-phospho-(1′-rac-glycerol) (sodium salt) (DMPG) from second harmonic generation (SHG) spectroscopy, quartz crystal microbalance with dissipation monitoring (QCM-D) and nanoplasmonic sensing (NPS) mass measurements, and atomistic simulations. The combined SHG/QCM-D/NPS approach provides interfacial charge density estimates from mean field theory for the attached peptides that are smaller by a factor of approximately two (0.12 ± 0.03 C m^–2^ for Lys_8_ and 0.10 ± 0.02 C m^–2^ for Arg_8_) relative to poly-l-lysine and poly-l-arginine. These results, along with atomistic simulations, indicate that the surface charge density of the supported lipid bilayer is neutralized by the attached cationic peptides. Moreover, the number of charges associated with each attached peptide is commensurate with those found in solution; that is, Lys_8_ and Arg_8_ are fully ionized when attached to the bilayer. Computer simulations indicate Lys_8_ is more likely than Arg_8_ to “stand-up” on the surface, interacting with lipid headgroups through one or two sidechains while Arg_8_ is more likely to assume a “buried” conformation, interacting with the bilayer through up to six sidechains. Analysis of electrostatic potential and charge distribution from atomistic simulations suggests that the Gouy–Chapman model, which is widely used for mapping surface potential to surface charge, is semi-quantitatively valid; despite considerable orientational preference of interfacial water, the apparent dielectric constant for the interfacial solvent is about 30, due to the thermal fluctuation of the lipid–water interface.

## Abstract 2

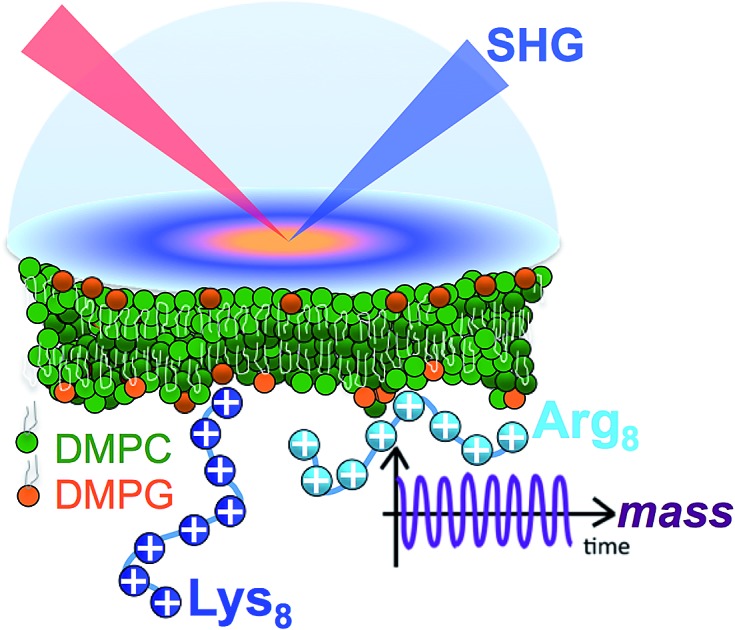
Quantifying the number of charges on peptides bound to interfaces requires reliable estimates of (i) surface coverage and (ii) surface charge, both of which are notoriously difficult parameters to obtain, especially at solid/water interfaces. Here, we report the thermodynamics and electrostatics governing the interactions of l-lysine and l-arginine octamers (Lys_8_ and Arg_8_) with supported lipid bilayers prepared.

## I. Introduction

Peptides and their interactions with biological and engineered membranes are of importance in the development of antimicrobial surfaces and therapeutics,^1^–^6^ non-viral vectors,^7^–^12^ layer-by-layer thin films,^13^–^15^ and for understanding the progression of many neurodegenerative diseases.^16^,^17^ Cell penetrating peptides containing cationic amino acids such as arginine and lysine can be used to cross cell membranes^18^ and deliver compounds to the cell interior by exploiting the surface charge of biological membranes.^9^ Small peptides also provide an opportunity to probe how short segments of polycations may interact with surfaces, with direct relevance to understanding how engineered nanomaterials, often manufactured with polycationic wrappings, interact with their environment.^19^,^20^ Despite the importance of charge density in these cases, a need remains to quantify polypeptide/membrane interactions from a perspective of the number of charges present at interfaces and the extent to which these charges are subject to contact ion pairing or p*K*_a_ shifts.^21^–^23^ Indeed, the ability to “count” the number of charges on peptides attached to solid or soft matter surfaces, especially in a label-free fashion, would represent a significant step towards understanding, controlling, and predicting peptide–surface interactions. However, doing so requires reliable estimates of (i) surface coverage and (ii) surface charge, both of which are notoriously difficult parameters to obtain at solid/water interfaces, especially if one wishes to avoid complications commonly associated with the use of external labels.

Recent mechanistic studies pairing molecular dynamic simulations and fluorescence assays have shown that arginine nonamers bind to lipid bilayers with a higher degree of cooperativity than do lysine nonamers,^21^ possibly explaining the cell-penetrating effectiveness of peptides containing arginine groups.^18^ Other studies of peptide interactions with lipid bilayers and cellular surfaces have reported on concomitant structural^2^,^24^–^26^ and electrostatic potential changes.^8^,^21^,^27^ The recent work by Cremer and co-workers employed a pH-sensitive fluorescence assay to provide qualitative and semi-quantitative information about the binding of lysine and arginine nonamers to supported lipid bilayers formed from zwitterionic (phosphatidylcholine, PC) and negatively charged (phosphatidylglycerol, PG) lipids on the basis of changes in local proton concentration upon nonamer adsorption.^21^ However, the surface charge density of the bilayer and those of the attached oligomers was not provided. To this end, the mass of the attached peptides and the interfacial charge density are necessary, albeit elusive, parameters which we focus on here.

**Scheme 1:**
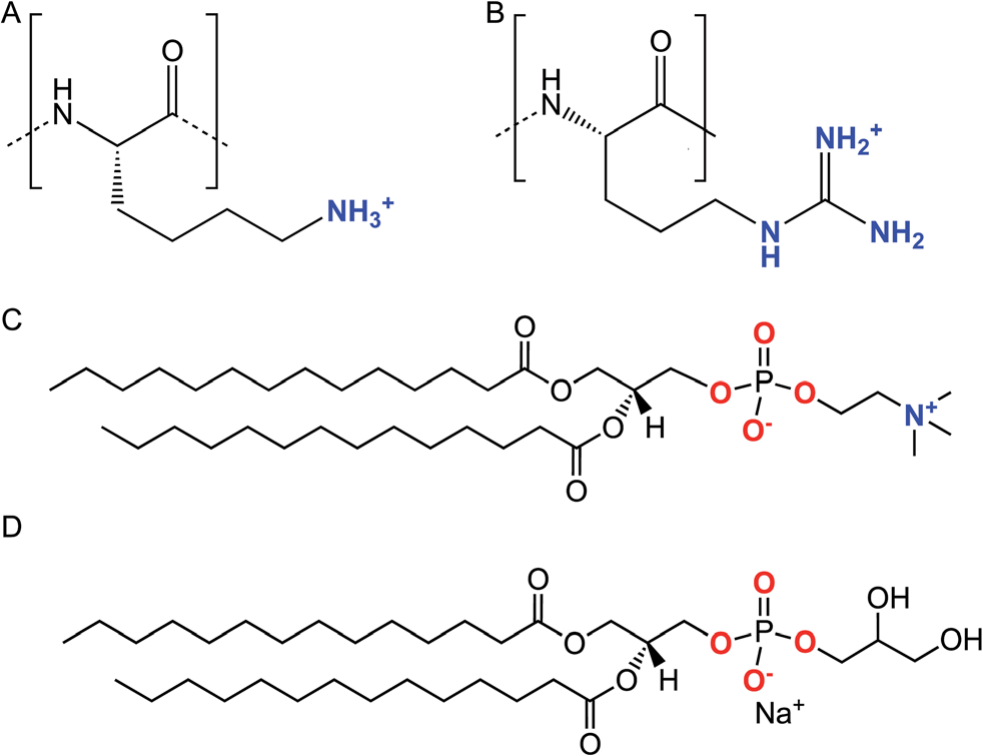
Chemical structures of the lysine (A) and arginine (B) repeat units in Lys_8_ and Arg_8_. Chemical structures of DMPC (C) and DMPG (D).

Our present work combines estimates of interfacial mass with nonlinear spectroscopic studies of interfacial electrostatics and atomistic simulations of octamers of lysine (Lys_8_) and arginine (Arg_8_) interacting with supported lipid bilayers used as idealized model systems mimicking some aspects of biological membranes (see Scheme 1). These peptides are amenable to investigations by atomistic computer simulations, which we employ to obtain further mechanistic information on the peptide–bilayer interactions.

We assess attached mass using acoustic and optical sensing techniques which are commonly employed to monitor the adsorption of biomacromolecules to surfaces,^28^–^33^ including cell surface models.^11^,^34^–^36^ Quartz crystal microbalance with dissipation monitoring (QCM-D) is used to obtain the acoustic mass of the attached oligomers (including the mass of dynamically coupled water).^29^ Simultaneous optical sensing by localized surface plasmon resonance spectroscopy (LSPR) yields estimates of the optical (solvent-free) mass of adsorbed species. Using this combined acoustic and optical sensing approach, we report on the water contents of oligomer films adsorbed to the supported lipid bilayer surface. The interfacial charge density and potentials and free adsorption energies are estimated by second harmonic generation spectroscopy (SHG), specifically using the Eisenthal *χ*^(3)^ method.^37^,^38^ This coherent nonlinear optical technique has been used previously to estimate interfacial charge densities and potentials, as well as free energies of adsorption and ion pairing/p*K*_a_ shifts for peptides,^8^ polycations,^39^ and anionic and cationic engineered nanoparticles^40^,^41^ and nanosheets^42^,^43^ interacting with model biological surfaces. Analysis of electrostatic potential and charge distribution from atomistic simulations helps evaluate mean-field models (*e.g.*, Gouy–Chapman)^44^ commonly used to map surface potential through the Eisenthal *χ*^(3)^ method to an apparent surface charge density, when the interface is rough at the molecular scale.

## II. Experimental methods

### A. Oligopeptide and lipid vesicle preparation

Lys_8_ and Arg_8_ were synthesized by and purchased from GenScript (Piscataway, NJ, purity ≥ 98%). Powders of Lys_8_ and Arg_8_ were stored in sealed plastic vials at –20 °C prior to use and were used without further purification. Powders (∼50 mg) were dissolved in 50–100 μL of ultrapure water (>18 MΩ cm; GenPure Pro or Millipore, Thermo Scientific) containing 0.001 M NaCl and vortexed, producing solutions with concentrations on the order of hundreds of mM. Lower concentrations were achieved through serial dilution. These stock solutions were then covered with Parafilm and stored in glass vials or microcentrifuge tubes at 4 °C. Immediately before use, the appropriate volume of oligomer solution was diluted to the desired concentration in 0.1 M NaCl buffered to pH 7.4 with 0.01 M Tris.

Small unilamellar vesicles were prepared at 9 : 1 molar ratios from 1,2-dimyristoyl-*sn-glycero*-3-phosphocholine (DMPC, Avanti Polar Lipids) and 1,2-dimyristoyl-*sn*-glycero-3-phospho-(1′-rac-glycerol) (sodium salt) (DMPG, Avanti Polar Lipids) as previously described.^45^,^46^ Here, we made the following modifications to our previously published method: lipid films were reconstituted with 0.001 M NaCl buffered to pH 7.4 with 0.01 M Tris, vortexed, sonicated for 30 min, and subjected to three freeze–thaw cycles by freezing in liquid N_2_ and thawing in a bath sonicator. Lipids were mechanically extruded as previously described.^45^,^46^ Immediately before use, extruded lipids were diluted to 0.5 mg mL^–1^ for SHG experiments or 0.125 mg mL^–1^ for QCM-D/NPS experiments in solutions composed of 0.1 M or in 0.15 M NaCl containing 0.005 M CaCl_2_, and buffered to pH 7.4 with 0.01 M Tris. Briefly, SLBs were formed *via* the vesicle fusion method. Specific experimental details are provided in the ESI.† For reference, all experiments described in the remainder of the article employed solutions buffered to pH 7.4 with 0.01 M Tris in the presence of 0.1 M NaCl.

### B. Determination of Lys_8_ and Arg_8_ surface mass densities

Our combined QCM-D and NPS setup allows us to simultaneously determine acoustic and optical surface mass densities of the lipid bilayers and the attached peptides. QCM-D measures changes in the resonance frequency (Δ*f*_*ν*_) and energy dissipation (Δ*D*_*ν*_) of the fundamental frequency and odd harmonics (*ν* = 3–11) of a (coated) AT-cut piezoelectric quartz crystal caused by interaction with an analyte.^47^ The acoustic mass sensed by QCM-D includes the mass of the analyte and that of any dynamically coupled solvent. The model used to estimate acoustic mass from the QCM-D frequency and dissipation response depends on the nature of the adlayer and the dissipation response.^48^ For rigidly coupled adlayers (taken as those with Δ*D*_*ν*_/(Δ*f*_*ν*_/*ν*) ≪ 0.4 × 10^–6^ Hz^–1^),^48^ the acoustic surface mass density (Δ*Γ*_QCM-D_) can be estimated here for the supported lipid bilayers from the Sauerbrey relation:^48^,^49^
1
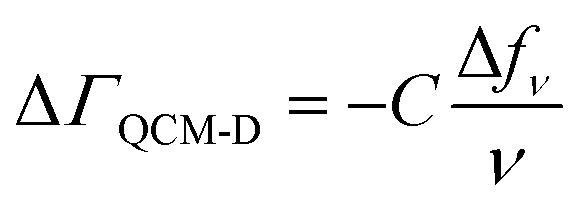
where *C* is the mass sensitivity constant (18 ng cm^–2^ Hz^–1^ at the fundamental frequency, *f*_1_ = 4.95 MHz used here) and depends on the properties of the quartz crystal. In the present study, the Sauerbrey relation was used to estimate acoustic surface mass densities of the supported lipid bilayers and octapeptide adlayers on the Si_3_N_4_-coated sensors. For more dissipative (*i.e.*, Δ*D*_*ν*_/(Δ*f*_*ν*_/*ν*) > 0.4 × 10^–6^ Hz^–1^), laterally homogeneous adlayers (in this study, the octapeptides on the supported lipid bilayers), acoustic mass can be estimated from the frequency and dissipation response for multiple harmonics using a Kelvin–Voigt model.^48^,^50^

Nanoplasmonic sensing relies on the sensitivity to changes in local refractive index of the localized surface plasmon resonance produced by illumination of noble metal nanoparticles.^51^ In the combined QCM-D/NPS setup employed, an array of nanoplasmonic gold discs embedded in the QCM-D sensor coating are illuminated in reflection mode and changes in the wavelength of maximum extinction (Δ*λ*_max_) are monitored. The de Feijter equation^52^ is used here to estimate optical surface mass density (Δ*Γ*_LSPR_) from Δ*λ*_max_:^53^2
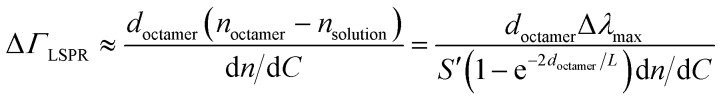
where *d*_octamer_ is the octamer adlayer thickness, *n*_octamer_ and *n*_solution_ are the refractive indices of the octamer and the solution, *S*′ is the refractive index sensitivity in the presence of the supported lipid bilayer, *L* is the decay length of the evanescent field, and d*n*/d*C* is the refractive index increment of the analyte at the LSPR wavelength. The optical surface mass density does not include contributions from the solvent. Further details on estimating Δ*Γ*_LSPR_ from Δ*λ*_max_ are provided in the ESI.†

The combined QCM-D/NPS setup was used to investigate the association of Lys_8_ and Arg_8_ with supported lipid bilayers formed *via* vesicle fusion from 9 : 1 DMPC/DMPG vesicles as described in the ESI.† For the Lys_8_ and Arg_8_ adlayers on supported lipid bilayers, Δ*D*_*ν*_/(Δ*f*_*ν*_/*ν*) values did not satisfy the Sauerbrey relation (Δ*D*_*ν*_/(Δ*f*_*ν*_/*ν*) ≪ 0.4 × 10^–6^ Hz^–1^). We therefore calculated acoustic masses of Lys_8_ and Arg_8_ adlayers on supported lipid bilayers using the Kelvin–Voigt viscoelastic model^50^ as implemented in QTools software (Version 3.0, Biolin Scientific). The bulk liquid phase was treated as a Newtonian fluid with a density of 1000 kg m^–3^ and dynamic viscosity of 0.001 kg m^–1^ s^–1^. Details on the analysis of the NPS data are presented in the ESI.†

### C. SHG laser system and SHG *χ*^(3)^ theory

Our SHG system, experimental setup, and analysis of the *χ*^(3)^ datasets have been previously described.^39^,^45^ Briefly, incident light (800 nm, 0.5 W, 120 fs pulse duration, 80 MHz reparation rate, s-in/all-out polarization combination) is focused onto the fused silica/bilayer/water interface near the angle of total internal reflection. A brief overview regarding SHG from charged interfaces is provided in the accompanying ESI† and more detailed work is published elsewhere.^38^,^54^–^57^

SHG *χ*^(3)^ measurements lend insight into binding thermodynamics and electrostatics.^8^,^38^,^39^,^58^ The second harmonic process is a second-order nonlinear optical process and as such, is not generally allowed in centrosymmetric systems under the electric dipole approximation. SHG signal intensity is directly proportional to the electric field generated at the second harmonic as shown in eqn (3),3
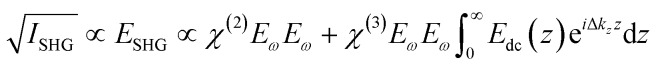
where *χ*^(2)^ and *χ*^(3)^ are the second- and third-order nonlinear susceptibility tensors, *E*_SHG_ is the electric field generated at the second harmonic, *E*_*ω*_ is the incident electric field oscillating at the fundamental frequency (800 nm), while *E*_dc_ is the *z*-(depth) dependent electric field produced by any interfacial charges. Integration yields the interfacial potential, *Φ*(*z* = 0), making the method useful as what is now termed an “optical voltmeter” for label-free probing of charged interfaces.^37^,^56^,^59^–^67^ In the reflection geometry employed here, and under the constant total electrolyte concentration of 0.1 M used, the inverse of the coherence length of the SHG process, Δ*k*_*z*_,^38^,^68^,^69^ is of such a magnitude that the SHG signal is produced close to the interface, minimizing the effect of phase matching.^8^,^57^,^61^,^70^,^71^

The SHG intensity from an initially negatively charged surface decreases upon cation adsorption, as the surface potential is rendered less negative, according to:

4 *E*_SHG_ ∝ *A* + *BΦ*_0_

where, *A* and *B* contain the second- and third-order macroscopic susceptibility of the interface, respectively, and the applied oscillating electromagnetic field from the incident laser, all of which are assumed to remain constant throughout, and *Φ*_0_ is 
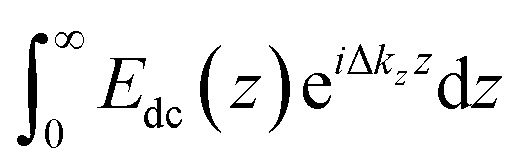
. The SHG adsorption isotherms were generated and analyzed largely as described previously,^39^,^72^ but employed the Hill model as discussed in the ESI.†

### D. Molecular dynamics (MD) simulations

To provide additional structural information on the peptide/bilayer interaction sites and the conformations assumed by the peptides upon attachment, atomistic molecular dynamics simulations were conducted using the CHARMM36 (ref. 73–75) force field to investigate Lys_8_ and Arg_8_ interacting with either a DMPC or a 9 : 1 DMPC/DMPG lipid bilayer; note that the NBFIX^76^ modification to the Lennard-Jones interactions associated with ions was applied to ensure proper description of ion adsorption to anionic lipids. Additionally, the simulations provided guidance to the analysis of interfacial charge density based on surface potential. The lipid bilayers were prepared with the CHARMM-GUI^77^ input generator. Each system had a starting dimension of 10 × 10 × 10 nm^3^, consisting of 288 DMPC, 32 DMPG and 36 peptide octamers. This initial peptide : lipid ratio was chosen based on our previous study of poly-allylamine hydrochloride (PAH) binding to the same lipid bilayer systems. Moreover, having multiple peptides adsorbed onto the lipid bilayer facilitated the exploration of the conformation that the peptides adopt upon adsorption. After neutralization, 0.15 M KCl was added to each system, and the system was solvated with TIP3P water. The NAMD^78^ and OpenMM^79^,^80^ packages were used for production runs on CPU and GPU, respectively; input files generated by CHARMM-GUI were used to ensure consistency among simulations using different hardware and software. For the NAMD simulations, the particle mesh Ewald (PME)^81^ method was applied with a grid size of 108, 108 and 90 for *X*, *Y*, and *Z* dimensions, respectively. For the peptides, each production run was executed over 500 ns. To evaluate the interfacial electrostatics, additional simulations (50 ns for production) were run for the 9 : 1 DMPC/DMPG system in which the *z*-dimension of the box was expanded to 18 nm so as to ensure a proper bulk region in the simulation box. Peptides that dissociated from the surface during the small-box simulations were removed in the large-box simulations (the new systems contain 16/5 Arg_8_/Lys_8_, leading to a surface binding density consistent with experimental measurements, see below), and the number of ions was adjusted accordingly based on the salt concentration of 0.15 M NaCl; weak harmonic positional restraints (with a force constant of 10 kJ nm^–2^) were applied to the amine nitrogen of the Lys sidechains that were bound to the lipid phosphate by the end of the production run for the small-box simulations to prevent any further peptide dissociation in the large-box simulations. The temperature for production run was set to 303.15 K, and NPT ensemble is applied with the *x*, *y* dimensions kept constant for all the systems studied here. For the numerical details for the analysis of interfacial electrostatic potential and charge density, see the ESI.†

## III. Results and discussion

### A. Peptide mass adsorbed to bilayers formed from 9 : 1 DMPC/DMPG

**Fig. 1:**
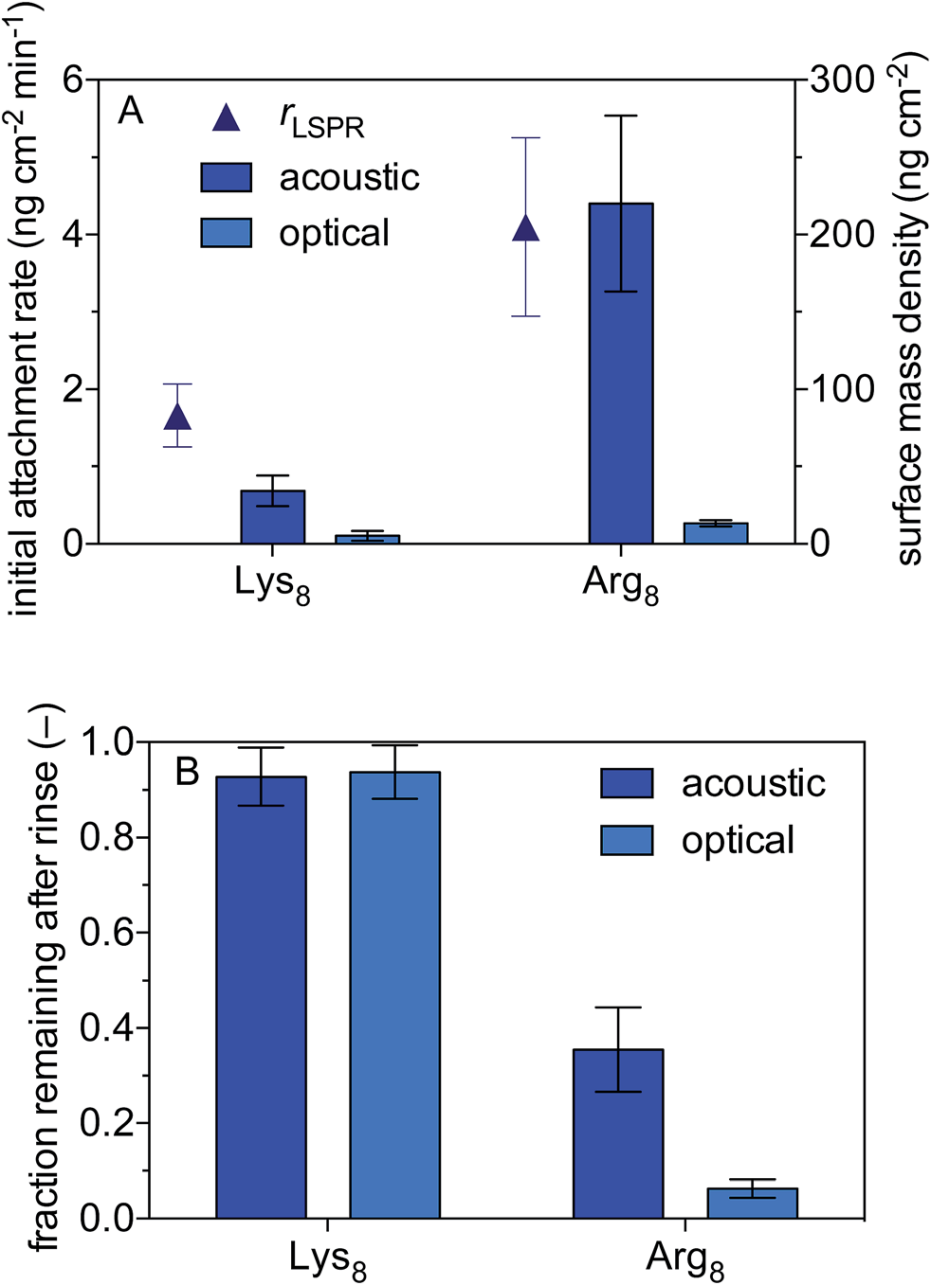
Attachment of octamers of lysine (Lys_8_) and arginine (Arg_8_) to supported lipid bilayers formed from 9 : 1 DMPC/DMPG. (A) Initial attachment rates and maximum acoustic and optical surface mass densities. The initial attachment rates were based on optical masses calculated from localized surface plasmon resonance data. (B) Acoustic and optical surface mass densities after 10 min rinse with oligomer-free solution. Solutions were 0.10 M NaCl buffered to pH 7.4 with 0.01 M Tris. Error bars represent one standard deviation of triplicate measurements.

We use a combined QCM-D and NPS setup to examine the initial rates of Lys_8_ and Arg_8_ attachment to supported lipid bilayers formed from 9 : 1 DMPC/DPMG, the surface mass densities of octapeptides adsorbed onto the supported lipid bilayers, and the extent of reversibility (Fig. 1). Representative QCM-D and LSPR traces are presented in Fig. S2 and S3,† respectively. The initial rate of octapeptide attachment to 9 : 1 DMPC/DMPC bilayers was larger for Arg_8_ than for Lys_8_ by a factor of 2.5 ± 0.9 (Fig. 1A) indicating that Arg_8_ had a higher affinity for the bilayer than did Lys_8_. Likewise, the maximum acoustic and optical surface mass densities attained by Arg_8_ were larger than those of Lys_8_ (Fig. 1A, Table 1). The maximum optical surface mass densities of Lys_8_ and Arg_8_ correspond to 3 ± 0.3 × 10^12^ and 5.7 ± 0.63 × 10^12^ molecules per cm^2^. If we assume that the peptides laid down individually on the bilayer, approximately 12 ± 1.3% and 27 ± 3.0% of the bilayer surface would be occupied by Lys_8_ and Arg_8_, respectively. Calculation details are provided in the ESI.†

**Table 1:** Summary of experimental data

	Binding constant, *K*_ads_ [×10^6^ M^–1^]	Adsorption free energy, Δ*G*_ads_ [kJ mol^–1^]	Surface charge density, *σ* [C m^–2^]	Hill coefficient, *n*	Acoustic^*a*^ and optical mass density [ng cm^–2^]	% ionization
Arg_8_	1.6 ± 0.4	–45 ± 1	0.10 ± 0.02	0.54 ± 0.09	210 ± 56	∼100
					13 ± 2	
Lys_8_	1.0 ± 0.3	–44 ± 1	0.12 ± 0.03	0.52 ± 0.09	34 ± 10	∼100
					5 ± 3	

^*a*^Maximum adsorbed acoustic mass at from viscoelastic modeling of 7^th^, 9^th^, 11^th^ harmonics prior to rinsing with peptide-free buffer.

LSPR measurements indicate that dynamically coupled water contributes considerably to the overall acoustic mass estimated by QCM-D for both peptides. In fact, a comparison of the acoustic and optical surface mass densities for Lys_8_ and Arg_8_ reveals that 83 ± 11% and 93 ± 3% of the acoustic masses is attributable to dynamically coupled water, respectively (Table S1†). Studies that employ both acoustic and optical sensing techniques find similarly high water contents for amino acids or amino acid-rich polymers adsorbed to surfaces.^82^,^83^ The large number of coupled water molecules associated with the highly charged Arg_8_ is likely a consequence of solvation effects which help to stabilize Arg–Arg pairs at the interface.^84^

After 10 min of rinsing with peptide-free buffer, we find that the attachment of both peptides is partially reversible over experimental time scales (Fig. 1) with Arg_8_ attachment being more reversible than that of Lys_8_. The Arg_8_ adlayer remaining after rinsing was more dissipative (Δ*D*_7_/(–Δ*f*_7_/7) = 2.0 ± 0.5 × 10^–6^ Hz^–1^) and had a higher water content (99 ± 0.8%) than that of Lys_8_ (Δ*D*_7_/(–Δ*f*_7_/7) = 0.02 ± 0.3 × 10^–6^ Hz^–1^; water content 82 ± 12%).

### B. Interfacial free binding energy and cooperativity

**Fig. 2:**
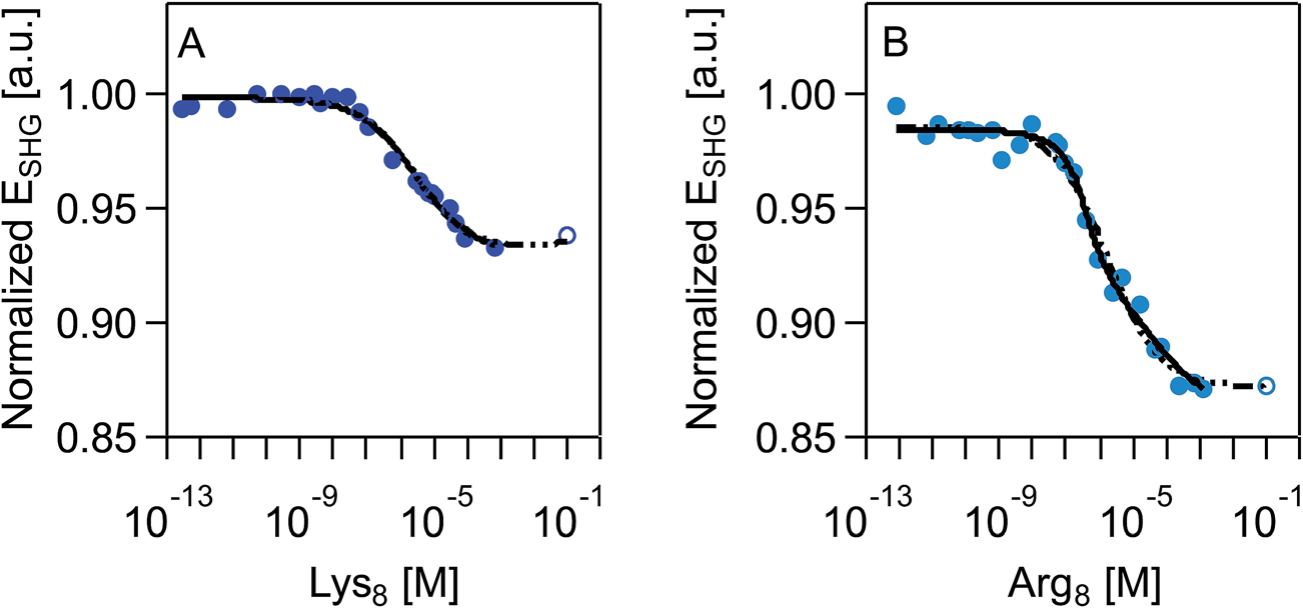
Normalized SHG *E*-field as a function of polymer concentration, in molarity, at 0.1 M NaCl, 0.01 M Tris, pH 7.4 for (A) Lys_8_ and (B) Arg_8_. Data sets include an extrapolated data point (shown as an open circle) that is the average of the last three measured data points. SHG *E*-field is normalized to the signal intensity associated with the supported lipid bilayer formed from 9 : 1 DMPC/DMPG prior to exposure to oligomers. Each individual adsorption isotherm is shown with the corresponding fit with the combined Hill/Gouy–Chapman equation applied to the acquired data (black solid line) and complete data set with extrapolated data point (dashed black line). See main text for further discussion.

SHG adsorption isotherms for Lys_8_ and Arg_8_ were recorded by exposing supported lipid bilayers formed from 9 : 1 DMPC/DMPG to increasingly higher concentrations of the respective octamers. An expected outcome of these surface potential-sensitive SHG experiments is that the observed SHG signal intensity decreases as the concentration, and thus the surface coverage, of the cationic Lys_8_ and Arg_8_ increases. Fig. 2 shows that this response is indeed observed. Moreover, we observe a nearly 10% larger decrease in the SHG *E*-field in the case of Arg_8_ than Lys_8_, suggesting that the adsorption of Lys_8_ results in a smaller change in interfacial potential than Arg_8_. This observation is in line with previous experimental^21^ and theoretical^85^ studies of these systems, which indicate that the interfacial potential (*i.e.*, surface charge) does indeed decrease (becoming more positive) upon exposure to lysine and arginine oligomers.

**Fig. 3:**
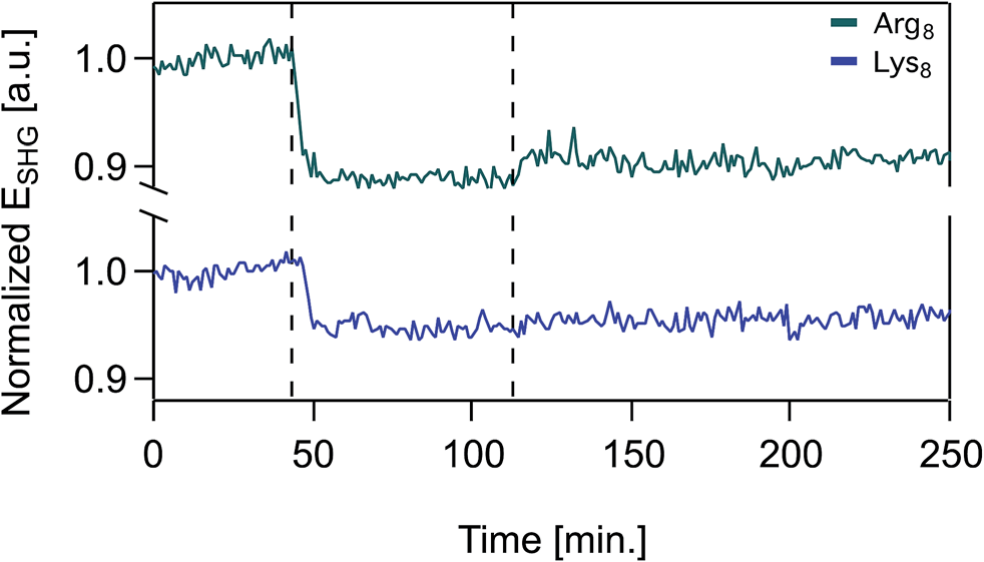
Normalized SHG *E*-field as a function of time in the presence of supported lipid bilayers formed from 9 : 1 DMPC/DMPG for 50 μM Arg_8_ (top trace, green), 50 μM Lys_8_ (bottom trace, blue) at 0.1 M NaCl, 0.01 M Tris, pH 7.4. At *t* = 0, the supported lipid bilayer is unperturbed and the SHG signal is monitored at 0.1 M NaCl. At *t* = 43 min, oligomer solution is introduced into the flow cell and at *t* = 112 min the flow cell is rinsed with oligomer-free solution composed of otherwise identical composition.

Over the timescales of our experiments, Lys_8_ and Arg_8_ interaction with the bilayer is partially reversible, as determined by SHG reversibility studies (Fig. 3 and S1A†) and confirmed by QCM-D mass estimates during (Fig. 1) and after exposure (ESI†). Yet, we cannot rule out that at *t* = ∞, the binding events are fully reversible. We therefore analyzed our adsorption isotherms, described next, with the caveat that full reversibility is not observed over the timescales of our experiments.

We fit our SHG adsorption isotherms using the combined Gouy–Chapman and Hill models we have used previously for polycations^39^ to obtain estimates (Table 1) of apparent equilibrium constants, charge densities, and Hill coefficients. The latter parameter describes the degree of cooperativity (or the lack thereof). While our isotherms approach surface saturation, due to the limited amounts of peptide available to us, complete saturation is not reached. Fitting these datasets results in large errors in the apparent equilibrium constants and charge densities likely because of difficulty achieving sufficiently high concentrations (>0.01 M) in our SHG experiments. However, prior literature indicates that saturation coverage is reached in the mM peptide concentration regime.^21^,^86^ We therefore analyzed the SHG adsorption isotherms by including one extrapolated SHG signal intensity point estimate for a peptide concentration of 0.1 M. This approach yielded similar point estimates relative to the ones obtained from fitting the isotherms while excluding the SHG signal intensity estimated for 0.1 mM peptide concentration (ESI†).

In contrast to the study by Cremer and co-workers,^21^ who reported significant differences in the cooperativity of adsorption of Lys_9_ (*n* = 0.22) and Arg_9_ (*n* = 0.73) to lipid bilayers formed from 9 : 1 molar ratios of 1-palmitoyl-2-oleoyl-*sn*-glycero-3-phosphocholine (POPC) and 1-palmitoyl-2-oleoyl-*sn*-glycero-3-phospho-(1′-*rac*-glycerol) (sodium salt) (POPG) (and 0.5 mol% *ortho*-rhodamine B which impacted at least Arg_9_ adsorption to POPC bilayers), we find comparable Hill coefficients of ∼0.5 (Table 1) for Lys_8_ and Arg_8_. This result suggests that the adsorption process is not cooperative for either octapeptide. The large Hill coefficient reported by Cremer and co-workers for Arg_9_ may be attributable to differences in bilayer fluidity between POPC/POPG and the addition of *ortho*-rhodamine B studied in their work in contrast to the label-free DMPC/DMPG system used here. The authors speculated that the inability of the lysine peptide to penetrate the lipid headgroup region could contribute to the apparent anti-cooperativity of lysine.^21^ However, we note that lower *n* values may also be explained by molecular heterogeneity where the experimentally derived binding curve is actually composed of an ensemble of individual binding curves.^87^ Indeed, such heterogeneity could arise from a number of factors including those introduced by differences in bilayer phase (liquid *versus* gel crystalline phases) and propensity and favorability of the octamers to bind to PC and/or PG lipid headgroups.

Previous studies by McLaughlin and co-workers have reported that the incremental increase in the free energy of adsorption upon elongating Lys oligomers by one Lys residue is ∼5.9 kJ per mol per residue (similar results were noted for arginine).^86^,^88^ We therefore expect free binding energies of –5.9 kJ mol^–1^ × 8 = –47.2 kJ mol^–1^ for our octamers. Our experiments show the binding energy estimate from the isotherms is ∼–44 ± 1 kJ mol^–1^ and –45 ± 1 kJ mol^–1^ for Lys_8_ and Arg_8_, respectively. As the comparison to McLaughin's data shows, the octamers attached without much noticeable cooperativity or anti-cooperativity (2 kJ mol^–1^ destabilization for the octamers *vs.* the purely additive expectation value) in terms of the free energy of adsorption. We therefore interpret the Hill coefficients from our fits (0.5) to indicate not anti-cooperativity but instead structural heterogeneity at the interface (as observed in atomistic molecular dynamics simulations, see below), a common alternate reason for Hill coefficients smaller than unity.^87^,^89^

### C. Interfacial charge densities and number of charges per attached peptide

Fitting SHG adsorption isotherms yields charge density estimates for Lys_8_ and Arg_8_ of 0.12 ± 0.03 C m^–2^ and 0.10 ± 0.02 C m^–2^, respectively. A sensitivity analysis shows that the approach is robust, as varying the value of the SHG intensity estimated for the 0.1 M peptide concentrations by 10% results in comparable charge densities, ranging from 0.02–0.16 C m^–2^ and 0.02–0.2 C m^–2^ for Lys_8_ and Arg_8_, respectively (Fig. S1†). Based on our previous estimates for the charge density of supported lipid bilayers formed from 9 : 1 DMPC/DMPG on fused silica (–0.1 C m^–2^),^39^,^41^ Lys_8_ and Arg_8_ attachment to the bilayers appears to result in charge neutralization.

Using the calculated charge densities from our SHG adsorption isotherms and the mass estimates derived from QCM-D and LPSR measurements, we provide next the lower and upper limits for the fraction of ionizable groups that remain charged upon adsorption to the membrane. Optical mass estimates correspond to surface coverages of about 10^16^ peptides (both Lys_8_ and Arg_8_) per m^2^, or 0.04 ± 0.02 C m^–2^ and 0.08 ± 0.01 C m^–2^ for Lys_8_ and Arg_8_, respectively, assuming they are fully (eight-fold) charged.

Our estimates of charge densities from the LSPR data, assuming the attached peptides are fully ionized, and the SHG experiments vary by a factor of about 3 for Lys_8_. In the absence of more reliable point estimates for the interfacial charge densities obtained from fitting eqn (S1) to the SHG adsorption isotherms, we cannot comment further on this difference. Taken together though, the charge density estimates from LSPR and SHG suggest that attached Lys_8_ and Arg_8_ are fully ionized under our experimental condition, consistent with their high bulk solution p*K*_a_ value.^90^

### D. Bound conformations of peptides and interfacial electrostatics from atomistic simulations

**Fig. 4:**
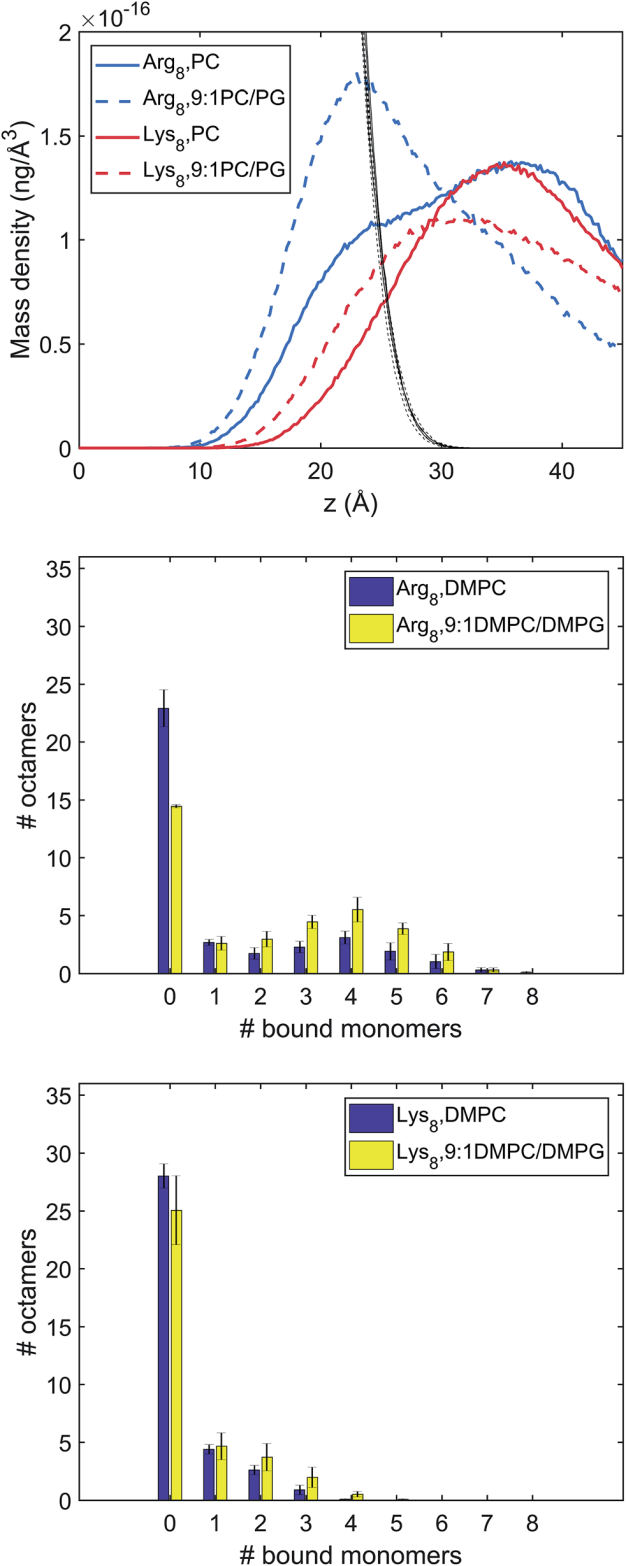
Characterization of different binding modes of Arg_8_ and Lys_8_ to the lipid membrane. Top: Mass density of peptide atoms along *z* (the membrane normal); the black lines indicate the mass density of lipid atoms. Middle and bottom panels: Distribution of the number of amino acid side chains bound to the lipid membrane (# of bound monomers) for the 36 copies of peptides in the simulation cell. The middle panel is for Arg_8_ and the bottom panel for Lys_8_. Evidently, Arg_8_ is observed to interact with the membrane through multiple sidechains, especially in the presence of anionic lipids; by contrast, Lys_8_ interact with the membrane with a small (1–3) number of sidechains.

To complement and provide additional insights for the experimental results, we performed MD simulations to explore Lys_8_ and Arg_8_ adsorption to lipid bilayers formed from DMPC and 9 : 1 DMPC/DMPG. With 36 octamers included in each system, the adsorption conformations of the octamers were extensively sampled. To investigate the conformations and number of bound octamers, we calculated the mass density and binding site distribution for the systems studied (Fig. 4). In our binding site analysis, a monomer is regarded as bound to the membrane surface if the representative atom of the charged group (CZ for arginine, NZ for lysine) is within the first peak of the corresponding radial distribution function (RDF) with respect to the lipid phosphorous atom; the corresponding distances are 5.5 and 4.5 Å for the arginine and lysine monomers, respectively.

**Fig. 5:**
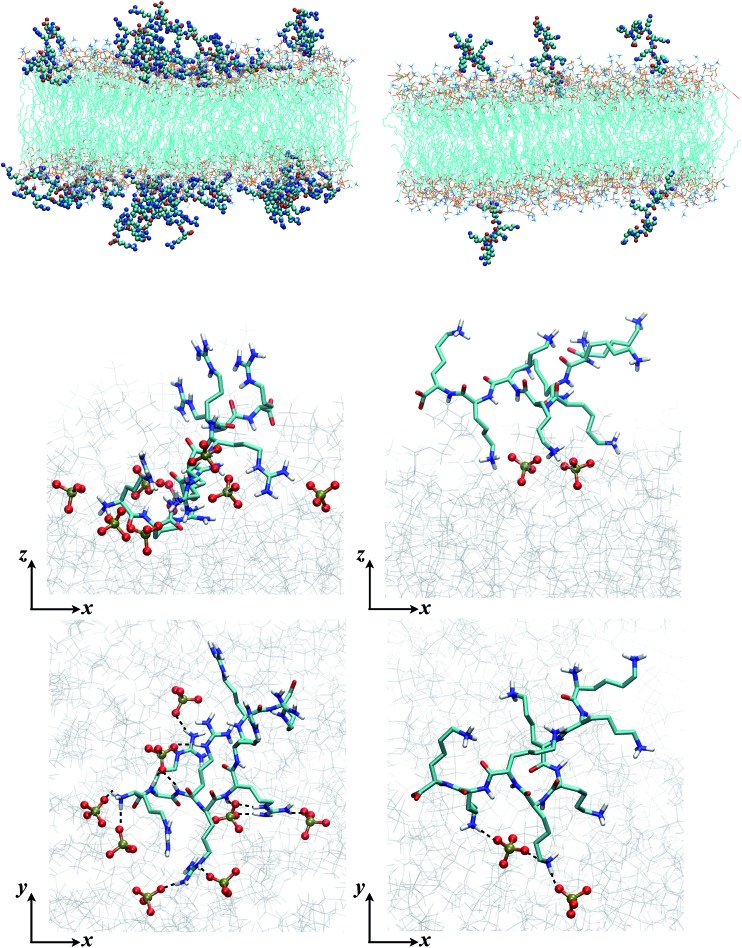
Snapshots from MD simulations (Arg_8_/Lys_8_ interacting with a 9 : 1 DMPC/DMPG bilayer) illustrate the different binding modes of the peptides. The left column is for Arg_8_, and right column for Lys_8_. The top two rows are sideviews (showing multiple peptides and a single peptide, respectively), which illustrate that due to the different numbers of sidechains interacting with the lipids, Lys_8_ peptides tend to point into the solution, while Arg_8_ peptides lay closer to the membrane; these trends are also illustrated by the mass density distributions (Fig. 4, top panel). The bottom row contains the top view of close-ups of the binding interactions; while Arg_8_ are engaged with multiple phosphate groups (those within 3 Å from Arg_8_ are shown in CPK), only a few lipid phosphate groups interact with the Lys sidechains.

The mass and binding site distributions (see Fig. 4) indicate that Arg_8_ and Lys_8_ bind to the lipid membrane with distinct affinities and conformations. The average number of bound octamers for Arg_8_ and Lys_8_ are 13.3 ± 1.6 and 8.4 ± 1.1 for the DMPC bilayer, respectively, while they are 22.6 ± 0.1 and 11.9 ± 3.0 for the 9 : 1 DMPC/DMPG bilayer, respectively. While each Lys_8_ peptide is most likely to bind with the bilayer through one or two sidechains with the bilayer, which is qualitatively similar to observations from previous atomistic MD simulations,^85^ Arg_8_ is most likely to attach to the bilayer *via* 3–5 binding sidechains (Fig. 5). Examination of these snapshots also reveals stacking of Arg sidechains^91^ from either the same peptide or neighboring peptides, and association of Arg_8_ mediated by the C-terminal carboxylate group, as discussed in recent studies.^92^,^93^ These observations indicate that while Lys_8_ is inclined to “stand-up” on the surface, Arg_8_ is likely to assume a “buried” conformation, as also evident from the mass density distribution. We note that the simulated bilayers differ from the supported lipid bilayers which are experimentally studied herein. Specifically, in our experimentally evaluated systems, the peptides are expected to approach the lipid bilayer from only one face (the other face is in contact with the solid support); in the simulations, peptides were allowed to interact with both surfaces of the lipid bilayer as a way to efficiently sample multiple peptide–membrane binding conformations.

An explicit binding free energy simulation of Arg_8_/Lys_8_ to the lipid bilayers was not pursued here. Considering the diverse binding modes of the peptides observed in the unbiased atomistic simulations, it is not straightforward to identify a simple collective variable (*e.g.*, the center-of-mass separation of the peptide and bilayer) that defines the bound state of the peptide while ensuring an extensive sampling of distinct peptide conformations. Nevertheless, the mass density and binding site distributions presented in Fig. 4 suggest that Arg_8_ tends to interact more strongly with lipid bilayers than Lys_8_, especially in the presence of anionic lipids which is in agreement with the conclusions drawn from other studies.^88^ This is also consistent with previous studies that highlighted the differences between Arg and Lys in terms of their interactions with lipid bilayers and impact on lipid structure and organization.^94^–^97^ As discussed in previous studies,^84^,^94^,^98^,^99^ the guanidinium group in the Arg sidechain is able to engage in multiple hydrogen bonding interactions with the phosphate and glycerol groups in lipids, while the amine group in Lys forms localized hydrogen-bonding interactions with phosphate; as a result, Arg is able to insert deeper into the bilayer than Lys, and poly-Arg forms multiple interactions with the lipids. For both peptides, the cationic sidechains preferentially interact with the anionic DMPG lipids compared to the zwitterionic DMPC lipids (see Fig. S6†). The similar apparent binding free energies for Arg_8_ and Lys_8_ obtained in our SHG studies likely reflects the notion that the probe depth in our SHG studies is on the order of several nanometers under the specific salt concentration, thus the amount of “bound” peptides may have included those in the interfacial region that are only weakly associated with the bilayer. Yet, we caution that the distance dependence in the SHG signal generation process from charged interfaces, which is subject to phase matching, is only now beginning to be understood.^38^,^54^,^55^,^57^,^69^

Despite a considerable number of peptides adsorbed on the lipid bilayer, the net charge distribution at the interface is rather small (the integrated charge density is less than 0.01 C m^–2^), due to the strong charge compensation by the salt ions and also oriented water molecules (see Fig. S4†). This observation is qualitatively consistent with the SHG estimated charge density, although a direct comparison is difficult since the adsorption densities in the simulation and experiment likely differ.

The microscopic simulations also provide an opportunity to evaluate the relationship between electrostatics (*e.g.*, surface potential) and charge distribution in the interfacial region. In particular, we are interested in the quantitative accuracy of the Gouy–Chapman model,^44^ which is widely used to map the measured interfacial electrostatic potential to an apparent surface charge density. In the Gouy–Chapman model, the solvent is treated as a dielectric continuum with the bulk dielectric constant and considers the electrostatic interaction between only the surface charge and salt ions. Such approximations are unlikely to be valid for the lipid–water interface, where water molecules are known be strongly oriented and thus contribute significantly to interfacial electrostatics.^100^,^101^

**Fig. 6:**
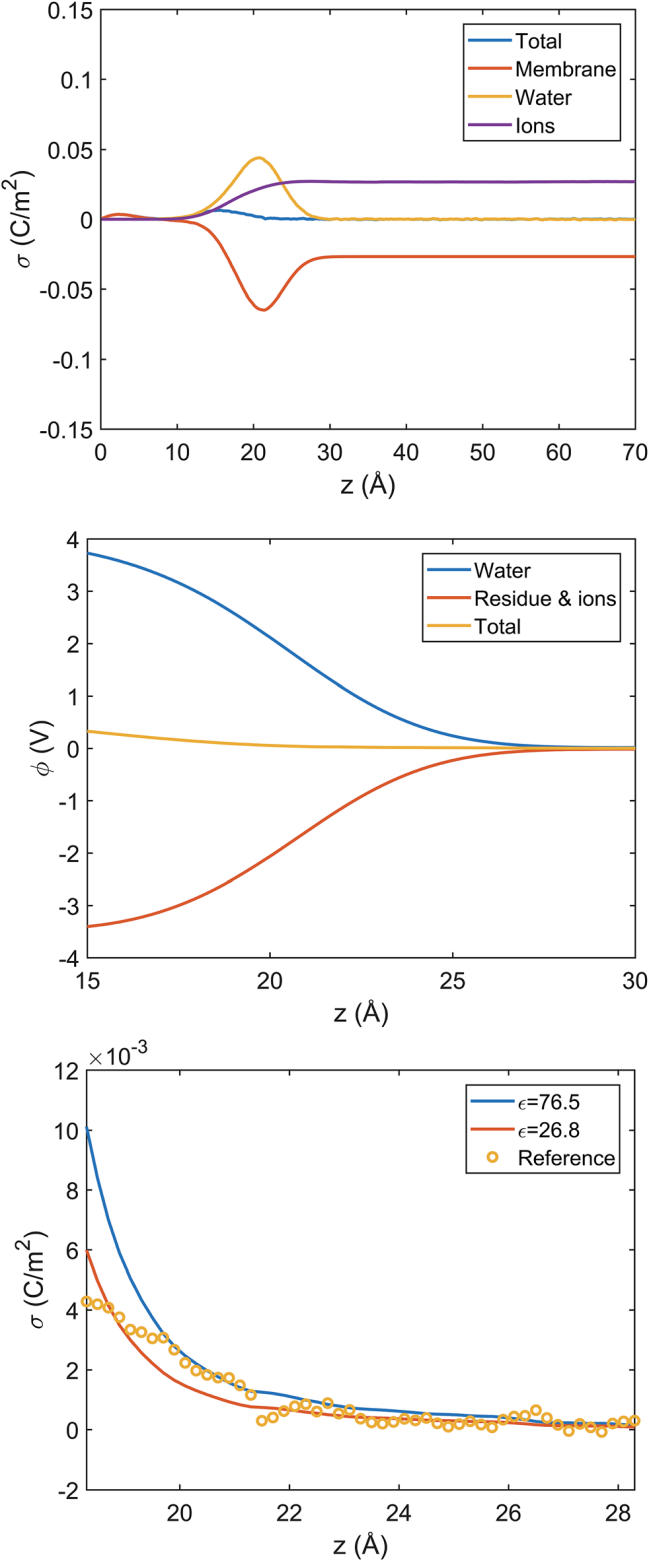
Analysis of charge distribution and electrostatic potential near the lipid/water interface for a 9 : 1 DMPC/DMPG bilayer; corresponding analyses for the bilayer with bound Arg_8_ and Lys_8_ peptides are discussed in the ESI.† Top: Integrated charge density from the center of the bilayer (*z* = 0), 
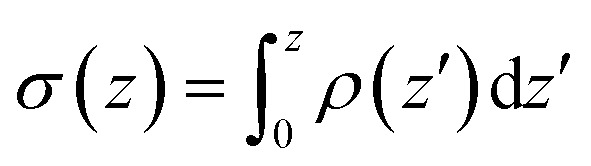
, where *ρ*(*z*′) is the charge density binned along *z* (the direction of the membrane normal) averaged over snapshots from MD simulations; note the significant contribution from interfacial water due to orientational preference. Middle: Electrostatic potential computed based on the charge density from MD simulations (eqn (13) in ESI†) illustrates a strong compensation between interfacial water and other components (lipids and salt ions). Bottom: Surface charge density computed with Grahame's equation and the electrostatic potential from MD simulations using different values of dielectric constant for the interfacial solvent. The open circles indicate integrated charge density from MD simulations (*i.e.*, the top panel). Since the precise location of the interface is not straightforward to determine, calculations based on the Grahame's equation are done for a series of *z* values near the location of the phosphate groups (*z* ∼ 20 Å). See ESI† for additional discussions.

Since systems with adsorbed peptides exhibit significant heterogeneity in the mass and charge distributions in the *x*, *y* dimensions (*e.g.*, see Fig. 5 top row), it is not straightforward to define an interface and conduct electrostatic analysis. Thus, we focus our electrostatic potential analysis on the 9 : 1 DMPC/DMPG system without any peptides. Due to preferential orientation of water at the interface, the integrated charge density from the lipid center to a given distance along the membrane normal (*z*) has considerable contributions from water (Fig. 6 top panel). As a result, the average electrostatic potential at the interface, *φ*(*z*), exhibits a strong compensation between interfacial water molecules and membrane/ions (Fig. 6 middle panel). Fitting the electrostatic potential (*φ*) and integrated surface charge density (*σ*) for a series of *z* values to the Grahame equation (see discussion in ESI†) leads to an apparent dielectric constant of 27 for the solvent at the bulk water/lipid bilayer interface. This value is considerably less than the bulk dielectric constant for water (∼78 at 300 K), which is expected due to the preferential orientation for interfacial water molecules at the bilayer surface. Nevertheless, the apparent dielectric constant we find here is also substantially larger than the value (∼6) used to compute differential charge capacitance for charged solid surfaces.^101^ This can be explained by the considerable thermal fluctuation of the lipid/water interface, which leads to a rather broad distribution of water orientation compared to the solid/water interface.^102^,^103^ Therefore, considering the uncertainty in the surface potential measured from SHG due to the assumption of a sharp interface, the current analysis suggests that the use of Gouy–Chapman model to map the surface potential to an apparent surface charge density is justified at a semi-quantitative level.

## IV. Conclusions

In conclusion, we have presented experiments and molecular dynamics simulations aimed at providing molecular insights into the thermodynamics and electrostatics that govern the interactions of octamers of l-lysine and l-arginine (Lys_8_ and Arg_8_) with supported lipid bilayers formed from 9 : 1 DMPC/DMPG. Comparison of acoustic and optical surface mass density estimates for Arg_8_ and for Lys_8_ indicate the presence of considerable amounts of dynamically coupled water. These interfacial water molecules, and how they respond to varying conditions of charge density due to peptide adsorption, can be probed using surface-specific vibrational spectroscopies, such as vibrational sum frequency generation (SFG),^104^,^105^ which is underway in one of our laboratories but with a specific emphasis on properly accounting for the interfacial potential-dependent *χ*^(3)^ contribution and the resulting absorptive–dispersive mixing with the *χ*^(2)^ contributions.^58^ SHG adsorption measurements sensitive to surface potential indicate that Lys_8_ and Arg_8_ attach without exhibiting noticeable cooperativity, as indicated by Hill coefficients of 0.5 ± 0.1 for both peptides. Yet, the binding free energies (–44 ± 1 kJ mol^–1^ and –45 ± 1 kJ mol^–1^ for Lys_8_ and Arg_8_, respectively) are purely additive when compared to those reported for arginine and lysine monomers. As such, the Hill coefficients found here are likely to report on interfacial heterogeneity, and not anti-cooperativity. Further, mass estimates from QCM-D and LSPR, and MD simulations suggest that Arg_8_ binds to a larger extent than Lys_8_. We find comparable equilibrium constants for both octapeptides by SHG (*n. b.* we did not determine *K*_ads_ by QCM-D or LSPR, due to the approximately 2 ng cm^–2^ sensitivity limit of QCM-D, which would prevent detection of sub-monolayer surface coverages needed to determine *K*_ads_ in this case). Yet, we caution that the *K*_ads_ point estimates were derived from the Langmuir-based adsorption model (Hill), whose assumptions (single site, monolayer limit, full reversibility) may not necessarily be applicable for our experimental conditions. In the molecular simulations, we found that Lys_8_ is more likely to “stand-up” on the bilayer surface, where it interacts through one to two sites, while Arg_8_ is more likely to assume a “buried” conformation, interacting with the bilayer through up to five sites. However, these simulations do not lead to a straightforward inference of the apparent “binding free energy” of Arg_8_*vs.* Lys_8_, which is a balance between interaction energy, number of dominant binding modes, and configurational entropy of the bound oligomer. These are the subjects of ongoing investigations by our groups.

The binding free energies for the peptides are about 10 kJ mol^–1^ smaller than those we recently reported for the polymeric counterparts poly-l-lysine (PLL) and poly-l-arginine (PLR).^37^ However, when we compute the free energy binding estimates for the octamers and polymers using charge, as opposed to molecular concentration, we find that this difference in binding free energy is considerably smaller (ESI Table S2†).

Upon accounting for the charge density of the bare bilayer, the attached peptides show an interfacial charge density that is approximately two times smaller (0.12 ± 0.03 C m^–2^ for Lys_8_ and 0.10 ± 0.02 C m^–2^ for Arg_8_) when compared to PLL and PLR. These results, and atomistic simulations, indicate that the surface charge density of the supported lipid bilayer is neutralized by the attached cationic peptides. Further, analysis of interfacial electrostatics and charge density based on the atomistic simulations supports that the Gouy–Chapman model used in the analysis of SHG data is appropriate at a semi-quantitative level, especially considering the subtleties associated with the *χ*^(3)^ approach. From our surface mass density estimates, we find that the number of charges associated with each attached peptide is commensurate with those found in buffer solution, *i.e.* Lys_8_ and Arg_8_ are fully ionized when attached to the bilayer, in contrast to the large range of ionization of the attached polycationic counterparts we reported earlier.^37^ Overall, the electrostatic, thermodynamic, and structural information reported here provides the opportunity to further understand, control, and predict the charge–charge interactions that govern peptide/membrane interactions at biological and engineered interfaces.

## Conflicts of interest

There are no conflicts to declare.

## Supplementary Material

Supplementary information
