# Supplementary material for: Counting charges on membrane-bound peptides
Source: Chem Sci. 2018 Apr 3;9(18):4285–98. doi: 10.1039/c8sc00804c (PMC5944241; doi:10.1039/c8sc00804c)
Supplement: Supplementary file 1 [file SC-009-C8SC00804C-s001.pdf]

Electronic Supplementary Material (ESI) for Chemical Science.
This journal is © The Royal Society of Chemistry 2018

Supporting Information for Counting Charges on Membrane-bound Peptides

Alicia C. McGeachy,[a†] Emily R. Caudill,[b†] Dongyue Liang,[b†]

Qiang Cui,[b,d] Joel A. Pedersen,[b,c,e,f] and Franz M. Geiger[a*]

[a]Department of Chemistry, Northwestern University, 2145 Sheridan Road, Evanston, IL 60660; [b]Department of Chemistry, University of Wisconsin-Madison, 1101 University Avenue, Madison, WI 53706; [c]Environmental Chemistry and Technology Program, University of Wisconsin-Madison, 660 North Park Street, Madison, WI 53706; [d]Department of Chemistry, Boston University, 590 Commonwealth Ave., Boston, MA 02215, USA; [e]Department of Soil Science, University of Wisconsin-Madison, 1525 Observatory Drive, Madison, WI 53706; [f]Department of Civil & Environmental Engineering, University of Wisconsin-Madison, 1415 Engineering Drive, Madison, WI 53706

I. Procedures for SHG Experiments

A. Substrate Preparation. UV-grade hemispherical silica lenses (1" diameter, ISP Optics) used in the SHG experiments were soaked with Nochromix® for at least 1 h and then rinsed copiously with ultrapure water. The lens was then transferred to a clean beaker and sonicated in methanol for 10 min, rinsed with ultrapure water, and dried under a stream of N_2. The hemispheres were then plasma cleaned for 10 min and stored in ultrapure water until use.

B. Bilayer Formation. After equilibrating the SHG flow cell with a solution composed of 0.15 M NaCl and 0.005 M $CaCl_2$, lipid vesicle suspensions were injected at a flow rate of 2 mL·min^{-1}. After allowing the bilayer to form over at least 15 min, the flow cell was flushed with 10 mL of 0.15 M NaCl + 0.005 M $CaCl_2$, 10 mL of 0.15 M NaCl, and finally 20 mL of 0.1 M NaCl. All SHG experiments were performed at room temperature (~21 °C). Fluorescence recovery after

photobleaching has shown that this method of vesicle preparation produces well-formed bilayers.[40, 46] All experiments were conducted in at least triplicate.

C. Experimental Procedure. When acquiring our SHG adsorption isotherms, the lowest oligomer concentration was introduced at a flow rate of 2 mL·min^{-1} in 0.1 M NaCl and allowed to interact for at least 15 min or until a steady SHG signal was achieved and followed by sequentially higher concentrations. Reversibility studies were also carried out, and in these studies the baseline at 0.1 M NaCl was acquired for at least 45 min. The supported lipid bilayer was then exposed to Lys_8 or Arg_8 at a concentration of 25 µM or 50 µM while maintaining the background electrolyte concentration. After ~70 min, the flow cell was then flushed with 0.1 M NaCl and the signal was monitored for at least 1 h.

D. Combined Gouy-Chapman/Hill Model. As discussed in our previous work,[1] using a combination of surface complexation models like the Gouy-Chapman model[2, 3] and Hill isotherms (*vide infra*) (Eq. S1) we are able to provide the lower estimates for charge densities and adsorption free energies arising from the interactions between the polypeptides and supported lipid bilayers.

$$E_{SHG} \propto A + B \sinh^{-1}\left(\left(\sigma_0 + \sigma_{ads}\left(\frac{K_{ads}^n M^n}{1 + K_{ads}^n M^n}\right)\right)\left(\frac{8.5\ M^{1/2} m^2 C^{-1}}{\sqrt{M + C_{elec}}}\right)\right) \quad \text{S1}$$

Here, $\sigma_0$is the charge density of the 9:1 DMPC/DMPG bilayer (– 0.1 C·m^{-2}),[1, 4] σ_{ads} is the charge density of the adsorbed polypeptide at monolayer coverage, K_{ads} is the apparent equilibrium, n is the Hill coefficient describing cooperativity of the adsorption process, M is the bulk peptide concentration, and C_{elec} is the background electrolyte concentration (0.1 M NaCl plus 0.008 M contribution from Tris) (T = ~20.5 °C). In addition to the reversibility studies at 50 µM discussed in the main text, we also completed SHG reversibility studies at 25 µM to match the concentrations used in the QCM-D and LSPR experiments discussed in the main text, and below (Figure S1A).

II. Dependence of Extrapolated Data Point on Estimated Charge Density from SHG Spectroscopy

As described in the main text, due to the expense of the synthesized oligomers of Lys and Arg and the volume that would we needed to achieve concentrations higher than ~10^{-3} M, we opted to use an artificial data point to estimate the lower bound for our reported charge density. This data point (shown as the open circle in Figure 3 in the main text) was determined by taking the average of the three data points at the highest Lys_8 and Arg_8 concentrations achieved experimentally. In similar studies of the adsorption of oligomers of Lys_8 and Arg_8 to SLBs, monolayer coverage is achieved at concentration on the order of 0.1 mM.[5] The extrapolated data point is placed at a concentration of 0.1 M Lys_8 or Arg_8. Varying the *y*-value (normalized SHG E-field determined by the average of the last three data points) by 10% results in charge densities that range from 0.02 to 0.16 $C{\cdot}m^2$ and 0.02 to 0.2 $C{\cdot}m^{-2}$ for Lys_8 and Arg_8 respectively. (Figures S1B and S1C)

III. Comparing SHG results for PLL/PLR to Lys_8/Arg_8

We have previously explored the adsorption of higher molecular weight polymers of L -lysine and L -arginine. In those studies, we found that the free energy of adsorption for both PLL and PLR was approximately –50 kJ $\cdot$moL^{-1}. Comparatively, we estimate free energies of adsorption for Lys_8 and Arg_8 of about –40 kJ $\cdot$moL^{-1}. If, however, we compare PLL/PLR to Lys_8/ Arg_8 on the basis of charge concentration instead of polymer concentration, we find that the difference in free energy between PLL/PLR and Lys_8/ Arg_8 is actually much smaller (Table S2). To determine the number of charged groups per polymer, we divided the average polymer concentration by the molar mass of the molar mass of either a lysine or arginine sub-unit (including mass contributions from the associated anion in the case of PLL and PLR. In the case of PLL and PLR, we used the combined Langmuir/Gouy-Chapman model instead of the Hill/Gouy-Chapman for reasons explained in our

previous work. Fitting the SHG E-fields versus polymer concentration or charge concentration does not change the overall charge density, demonstrating the robustness of the fitting models used.

IV. Combined QCM-D/NPS Experiments

A. Experimental Setup. The combined QCM-D/NPS experiments were conducted using a Q-Sense E4 instrument (Biolin Scientific, Göteborg, Sweden) equipped with a window module (QWM401) with an Acoulyte accessory (Insplorion AB, Göteborg, Sweden) placed atop the window cell and connected to the optics unit. The topographically flat Acoulyte sensors were QCM-D chips modified with an array of 100 nm diameter, 20 nm thick gold nanodisks embedded in the SiO_2 layer and coated with Si_3N_4 (10 nm) by plasma enhanced chemical vapor deposition.[12] The spectral acquisition frequency was 1 Hz; each recorded spectrum was the average of 200 spectra collected with an integration time of 0.3 ms. Acoulyte Si_3N_4-coated QCM-D/NPS sensors (Insplorion) were soaked in 2% (w/v) SDS for 10 min, rinsed sequentially with ethanol and ultrapure water, dried under N_2, and treated with UV/ozone for 15 min (Bioforce Nanosciences UV/Ozone Procleaner) to produce a thin SiO_2 layer immediately prior to mounting in the flow cell.

B. Bilayer Formation. To form supported lipid bilayers in the QCM-D/NPS setup, we first flowed a solution containing 0.10 M NaCl and 0.005 M $CaCl_2$ over the cleaned sensor until stable frequency, dissipation, and wavelength of maximum extinction (λ_{max}) readings were attained. Next, we flowed a vesicle suspension (0.125 mg·mL^{-1}) over the sensor for 15 min. The flow cell was then rinsed for 30 min with solution of the same composition as used to initially equilibrate the sensor. The flow cell was rinsed with $CaCl_2$-free buffer for at least 20 min, during which time the vesicles fused and ruptured, and a stable supported lipid bilayer was formed. After bilayer formation, we flowed 0.01 M NaCl over the supported lipid bilayer until stable frequency,

dissipation, and λ_{max} readings were obtained and then flowed 25 µM Lys_8 or Arg_8 over the bilayers in 0.10 M NaCl. After maximum peptide attachment had been reached as indicated by a plateau in frequency, dissipation, and λ_{max}, peptide-free 0.10 M NaCl solution was flowed over the bilayers for at least 30 min. In control experiments, we examined the association of Lys_8 and Arg_8 with the Si_3N_4-coated sensors, equilibrating the sensors in 0.10 M NaCl buffer prior to exposure to Lys_8 or Arg_8. In all QCM-D/NPS experiments, temperature was maintained at 23 ± 0.5 °C and the flow rate was 0.1 $mL\cdot min^{-1}$. All experiments were conducted in at least triplicate.

C. QCM-D and LSPR Sensor and Flow Cell Cleaning. The nanoplasmonic sensing module was removed and the connected instrument was turned off prior to cleaning. The temperature was raised to 40 °C using the QCM-D software. First, 10 mL of 2% sodium dodecyl sulfate (SDS) solution was flowed over the LSPR sensor. Then, the sensor was removed and replaced with a gold QCM-D sensor, and 10 mL of Hellmanex, Cobas, and ultrapure water were flowed sequentially through the window cell. All components were dried with N_2.

V. Bulk Refractive Index Sensitivities of the LSPR Sensors

To calculate optical mass from $\Delta\lambda_{max}$, sensitivities of the sensor were measured in the absence and presence of a supported lipid bilayer (S and S', respectively). These values were used to calculate the distance L over which the LSPR signal decays to $1/e$ of the induced evanescent field from the nanoplasmonic gold discs on the sensor. Increasing concentrations of glycerol (0-35 mass% in increments of 5 mass%) were measured in triplicate at 632.8 nm (Rudolph Research J157 Automatic Refractometer). These glycerol solutions were flowed over the LSPR sensor, and changes in frequency, dissipation, and $\Delta\lambda_{max}$ were recorded with both QCM-D and LSPR. These measurements were recorded for glycerol solutions before and after the presence of a bilayer. The $\Delta\lambda_{max}$ values were plotted against the measured refractive indices, and the slopes of the graphs

were linearly fitted to obtain S and S'. The effective refractive index within the LSPR sensing volume, n_{eff}, is then calculated for each replicate, where:

$$\Delta\lambda_{max} = S(n_{eff} - n_{solution}) \quad \text{S2}$$

and n_{buffer} is the refractive index increment of the solution measured in triplicate at 632.8 nm and determined to be 1.334 ± 0.00002 RIU.

VI. Calculation of Optical Mass from LSPR Data

The characteristic decay length L within the LSPR field was calculated from the bulk refractive index sensitivity values in Eq. S2, and determined to be 20 ± 3.3 nm via:

$$\frac{S'}{S} = e^{-\frac{2d_{SLB}}{L}} \quad \text{S3}$$

The bilayer thickness (d_{SLB}) was estimated from QCM-D measurements. Optical surface mass densities of octamer adlayers were estimated using the de Feijter equation:[6]

$$\Delta\Gamma_{LSPR} \approx \frac{d_{octamer}(n_{octamer} - n_{solution})}{dn/\,dC} \quad \text{S4}$$

where $n_{octamer}$ and $n_{solution}$ are the refractive indices of the octamer adlayer and the solution, the refractive index increments (dn/dC) were taken to be 0.177 ± 0.003 RIU·cm^3·g^{-1} for Lys$_8$ and 0.202 ± 0.003 RIU·cm^3·g^{-1} for Arg$_8$, which are generally accepted values for the monomer of each amino acid,[7, 8] and $d_{octamer}$ is the thickness of the octamer adlayer determined by QCM-D. For octamer adlayers on supported lipid bilayers, we determined $n_{octamer}$ from:[9, 10]

$$n_{octamer} = \frac{n_{eff} - n_{solution}}{1 - e^{-2d_{octamer}/L}} + n_{solution} \quad \text{S5}$$

and the effective refractive index, n_{eff}, was calculated from:

$$n_{eff} = \frac{\Delta\lambda_{max}}{S'} + n_{solution} = \frac{\Delta\lambda_{max}}{Se^{-2d_{SLB}/L}} + n_{solution} \quad \text{S6}$$

Inserting Eq. S5 and S6 into 4 yields the final formula for optical surface mass density, Γ_{LSPR}, for octamer adlayers on supported lipid bilayers:

$$\Gamma_{\mathrm{LSPR}} = \frac{d_{\mathrm{octamer}} \cdot \Delta\lambda_{\mathrm{max}}}{S\left(e^{-2d_{\mathrm{SLB}}/\mathrm{L}} - e^{-2(d_{\mathrm{SLB}}+d_{\mathrm{octamer}})/L}\right)\cdot dn/dC} = d_{\mathrm{octamer}} \frac{d_{\mathrm{octamer}} \cdot \Delta\lambda_{\mathrm{max}}}{S'(1 - e^{-2d_{\mathrm{octamer}}/L})\cdot dn/dC} \quad \text{S7}$$

Optical masses for the peptide directly deposited to the sensor were calculated using the following equation:

$$\Gamma_{\mathrm{LSPR}} = d_{\mathrm{octamer}} \cdot \frac{\Delta\lambda_{\mathrm{max}}}{S(1 - e^{-2d_{\mathrm{octamer}}/\mathrm{L}}) \cdot dn/dC} \quad \text{S8}$$

We estimated the surface coverage of octapeptides on the supported lipid bilayers based on the optical surface mass density, octapeptide molar mass, the active area of the sensor, and the projected area of the octapeptides assuming the peptides laid down individually. This orientation allowed us to place an upper bound on the fraction of the SLB surface area covered by the octapeptides. Projected areas were based on the dimensions of the octapeptides optimized in PyMOL.[11] The dimensions of Lys_8 and Arg_8 were respectively 2.76 nm × 1.45 nm and 2.77 nm × 1.71 nm.

VII. Analysis of Interfacial Electrostatics

The spatial charge distribution is averaged over the *x,y* plane and binned (with a width of δ = 0.2 Å) according to the value of *z* coordinate,

$$\rho(z) = \frac{1}{L\delta}\langle \sum_i q_i \delta(z_i - z)\rangle \quad \text{S9}$$

in which *L* is the area of the simulation cell in the *x,y* dimension, and the bracket indicates ensemble average (2,500 frames from the MD trajectories are used for each system); the bilayer center is set to be at *z* = 0. Plots of $\rho(z)$ for the systems analyzed are shown in Figure S4. The integrated charge density from the bilayer center up to a given *z* value is denoted as $\sigma(z)$:

$$\sigma(z) = \int_0^z dz' \rho(z') \tag{S10}$$

The electrostatic potential is calculated by noting from Gauss's law that,

$$\varphi(z) = \int_z^{+\infty} \frac{\sigma(z')}{\varepsilon_0} dz' = \frac{1}{\varepsilon_0} \int_z^{+\infty} \int_0^{z'} \rho(z")dz"\, dz' \tag{S11}$$

where ε_0 is the vacuum permittivity; when applying Gauss's law, the system is assumed to be symmetrical with respect to the bilayer center (thus the electric field at *z* is given by $E(z) = \sigma(z)/\varepsilon_0$, which is integrated from *z* to ∞ to obtain the electrostatic potential relative to the bulk). For a symmetrical system, this expression is equivalent to the double integration expression commonly used in the literature,[13, 14]

$$\varphi(z) = -\frac{1}{2\varepsilon_0} \int_{-\infty}^{z} \left[\int_{-\infty}^{z'} \rho(z")\mathrm{d}z" - \int_{z'}^{\infty} \rho(z")\mathrm{d}z" \right] dz' \tag{S12}$$

To apply the Gouy-Chapman model in the context of interpreting the SHG experiment, it is assumed here that the measurement senses the electrostatic potential due to all charges from the bilayer center up to a virtual interface located at *z*. Thus the electrostatic potential at this interface predicted by the Gouy-Chapman model and the integrated charge density are related through the Grahame's equation,[15]

$$\sigma(z) = \sqrt{8000cN_A\varepsilon_0\varepsilon_w k_B T}\sinh\left(\frac{ne\varphi_{GC}(z)}{2k_BT}\right) \tag{S13}$$

where *c* is the salt concentration in *M*, N_A is Avogadro's number, ε_w is the relative permittivity of the solvent, *n* is valence of salt ions (for the present case, *n*=1), *e* is the elementary charge, k_B is Boltzmann's constant, and *T* is temperature.

To evaluate the applicability of the Gouy-Chapman model to the lipid/water interface, electrostatic potential and integrated charge density computed from the microscopic MD simulations are best fitted to the Grahame's equation by adjusting the relative permittivity, ε_w,

which is expected to be different from the value for bulk solution.[16] Since the precise location of the interface is not straightforward to determine, the fitting was done for a series of *z* values close to the location of the lipid phosphate groups (see Figure 6 in the main text). Such analysis is not straightforward for the Arg_8/Lys_8 systems due to the significant heterogeneity in the computed electrostatic potential in the *x,y* plane (see Figure S5); therefore, the fitting to the Gouy-Chapman model was done only for the 9:1 DMPC/DMPG system.

References.

1. J. M. Troiano, A. C. McGeachy, L. L. Olenick, D. Fang, D. Liang, J. Hong, T. R. Kuech, E. R. Caudill, J. A. Pedersen, Q. Cui and F. M. Geiger, Quantifying the Electrostatics of Polycation–Lipid Bilayer Interactions, *J. Am. Chem. Soc.*, 2017, **139**, 5808-5816.
2. P. L. Hayes, J. N. Malin, D. S. Jordan and F. M. Geiger, Get Charged Up: Nonlinear Optical Voltammetry for Quantifying the Thermodynamics and Electrostatics of Metal Cations at Aqueous/Oxide Interfaces, *Chem. Phys. Lett.*, 2010, **499**, 183-192.
3. J. N. Malin, P. L. Hayes and F. M. Geiger, Interactions of Ca, Zn, and Cd Ions at Buried Solid/Water Interfaces Studied by Second Harmonic Generation, *J. Phys. Chem. C*, 2009, **113**, 2041-2052.
4. J. M. Troiano, L. L. Olenick, T. R. Kuech, E. S. Melby, D. Hu, S. E. Lohse, A. C. Mensch, M. Dogangun, A. M. Vartanian, M. D. Torelli, E. Ehimiaghe, S. R. Walter, L. Fu, C. R. Anderton, Z. Zhu, H. Wang, G. Orr, C. J. Murphy, R. J. Hamers, J. A. Pedersen and F. M. Geiger, Direct Probes of 4 nm Diameter Gold Nanoparticles Interacting with Supported Lipid Bilayers, *J. Phys. Chem. C*, 2015, ***119*** 534-546.
5. A. D. Robison, S. Sun, M. F. Poyton, G. A. Johnson, J. P. Pellois, P. Jungwirth, M. Vazdar and P. S. Cremer, Polyarginine Interacts More Strongly and Cooperatively than Polylysine with Phospholipid Bilayers, *J. Phys. Chem. B*, 2016, **120**, 9287-9296.
6. J. A. De Feijter, J. Benjamins and F. A. Veer, Ellipsometry as a Tool to Study the Adsorption Behavior of Synthetic and Biopolymers at the Air–Water Interface, *Biopolymers*, 1978, **17**, 1759-1772.
7. H. Y. Zhao, P. H. Brown and P. Schuckt, On the Distribution of Protein Refractive Index Increments, *Biophys. J.*, 2011, **100**, 2309-2317.
8. G. E. Perlmann and L. G. Longsworth, The Specific Refractive Increment of Some Purified Proteins, *J. Am. Chem. Soc.*, 1948, **70**, 2719-2724.
9. A. R. Ferhan, J. A. Jackman and N. J. Cho, Probing Spatial Proximity of Supported Lipid Bilayers to Silica Surfaces by Localized Surface Plasmon Resonance Sensing, *Anal. Chem.*, 2017, **89**, 4301-4308.
10. M. P. Jonsson, P. Jönsson and F. Höök, Simultaneous Nanoplasmonic and Quartz Crystal Microbalance Sensing: Analysis of Biomolecular Conformational Changes and Quantification of the Bound Molecular Mass, *Anal. Chem.*, 2008, **80**, 7988-7995.
11. L. Schrödinger, The PyMOL Molecular Graphics System, https://pymol.org/2/support.html).
12. F. A. A. Nugroho, R. Frost, T. J. Antosiewicz, J. Fritzsche, E. M. Larsson Langhammer and C. Langhammer, Topographically Flat Nanoplasmonic Sensor Chips for Biosensing and Materials Science, *ACS Sensors*, 2017, **2**, 119-127.
13. M. A. Wilson, A. Pohorille and L. R. Pratt, Study on the Liquid Vapor Interface of Water. 1. Simulation Results of Thermodynamic Properties and Orientational Structure - Comment., *J. Chem.Phys.*, 1989, **90**, 5211-5213.
14. I. Vorobyov and T. W. Allen, The Electrostatics of Solvent and Membrane Interfaces and the Role of Electronic Polarizability, *J. Chem.Phys.*, 2010, **132**, 185101.

15. J.-L. Barrat and J.-P. Hansen, *Basic Concepts for Simple and Complex Liquids*, Cambridge University Press, Cambridge, UK, 2003.
16. D. J. Bonthuis and R. R. Netz, Beyond the Continuum: How Molecular Solvent Structure Affects Electrostatics and Hydrodynamics at Solid-Electrolyte Interfaces, *J. Phys. Chem. B*, 2013, **117**, 11397-11413.

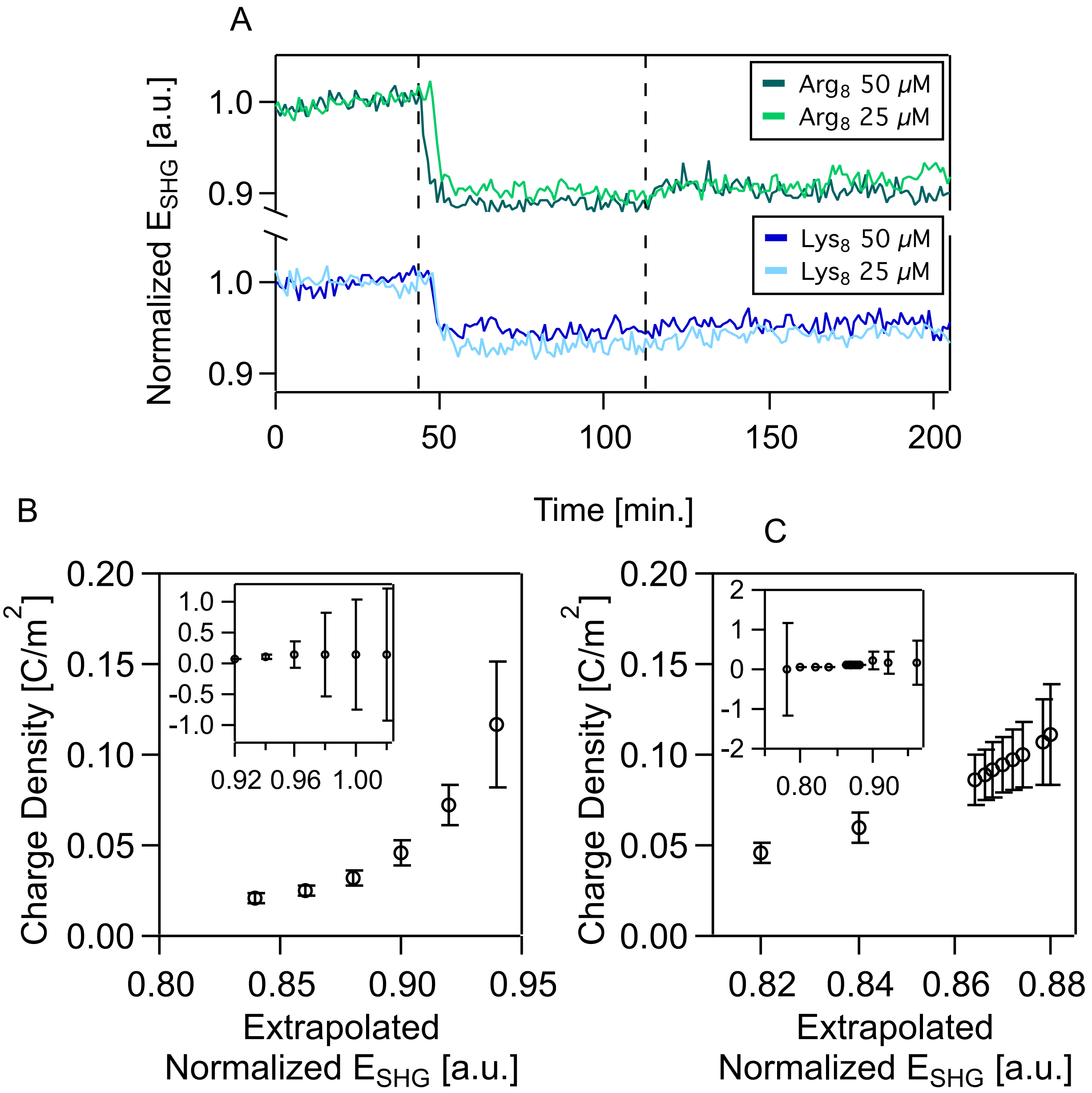

Figure S1. (A) Normalized SHG *E*-field as a function of time in the presence of supported lipid bilayers formed from 9:1 DMPC/DMPG for 25 µM and 50 µM (reproduced from Figure 3 in main text) octapeptide concentrations at 0.1 M NaCl, 0.01 M Tris, pH 7.4. At $t = 0$, the supported lipid bilayer is unperturbed and the SHG signal is monitored at 0.1 M NaCl. At $t = 43$ min, oligomer solution is introduced into the flow cell and at $t = 112$ min the flow cell is rinsed with oligomer-free solution composed of otherwise identical composition. Charge density of (B) Lys_8 and (C) Arg_8 adsorbed to an SLB formed from 9:1 DMPC/DMPG as a function of the normalized SHG *E*-field used for the extrapolated data point at 0.1 M NaCl. Insets are a zoomed-out view of the same data to highlight the large errors associated with charge density values at higher and lower extremes for the extrapolated signal intensity at 0.1 M octamer concentrations.

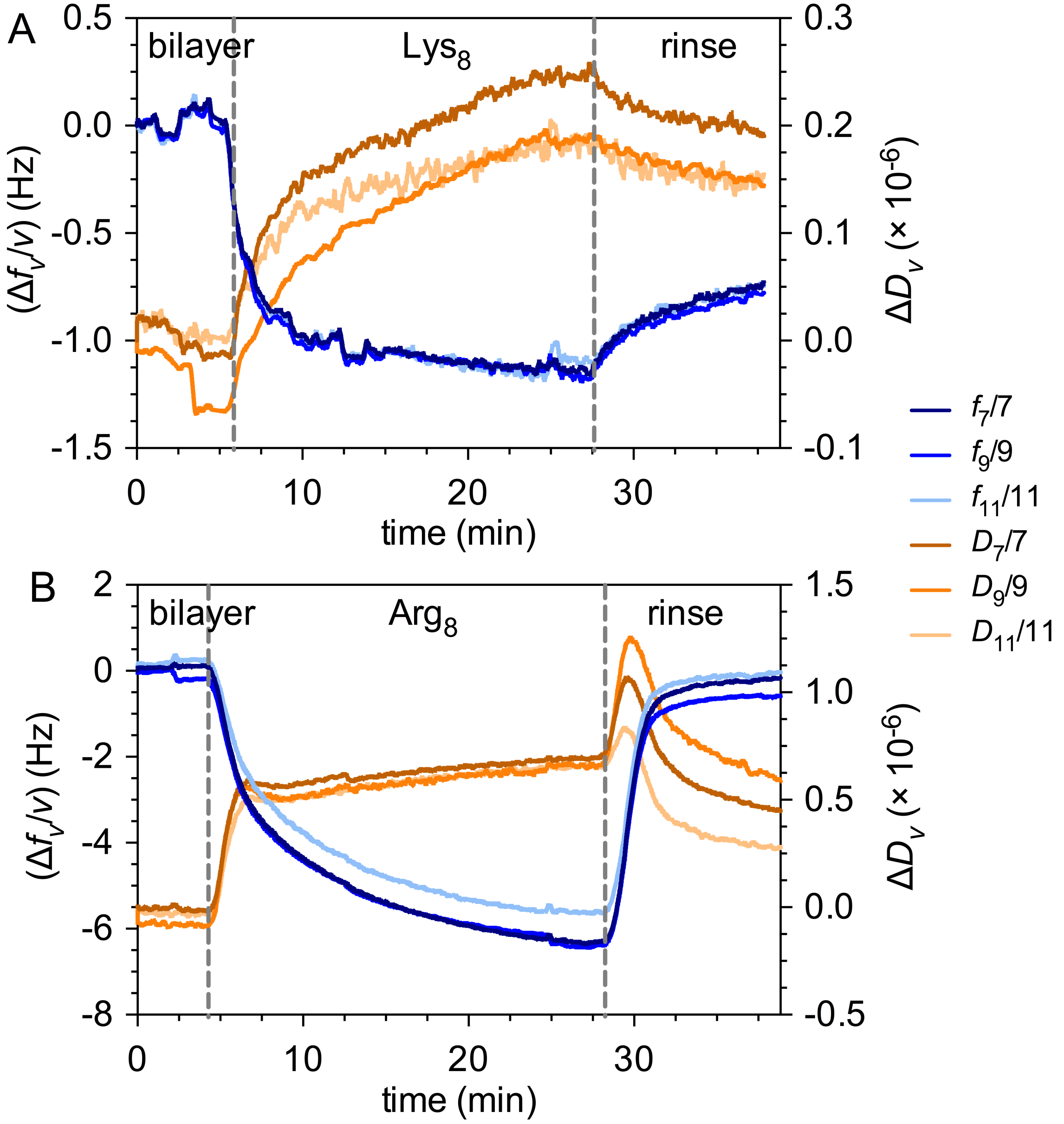

Figure S2. Representative QCM-D data recording (A) Lys_8 and (B) Arg_8 attachment to 9:1 DMPC/DMPG bilayer in 0.1 M NaCl buffered to pH 7.4 with 0.01 M Tris at 23 °C followed by rinse with polymer-free buffer. Concentrations of Lys_8 and Arg_8 were 25 µM. Changes in solution composition are indicated by arrows above and dashed lines in each plot. Traces were smoothed by 31-point boxcar averaging.

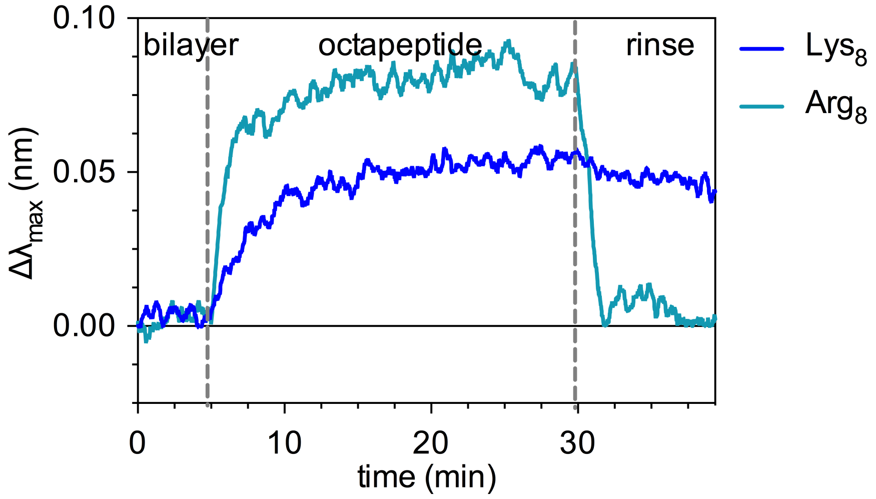

Figure S3. Representative NPS data recording Lys_8 and Arg_8 attachment to 9:1 DMPC/DMPG bilayer in 0.1 M NaCl buffered to pH 7.4 with 0.01 M Tris at 23 °C followed by rinse with peptide-free buffer. Concentrations of Lys_8 and Arg_8 were 25 μM. Changes in solution composition are indicated by arrows above and dashed lines in each plot. Traces were smoothed by 31-point boxcar averaging, and corrected for instrumental baseline drift.

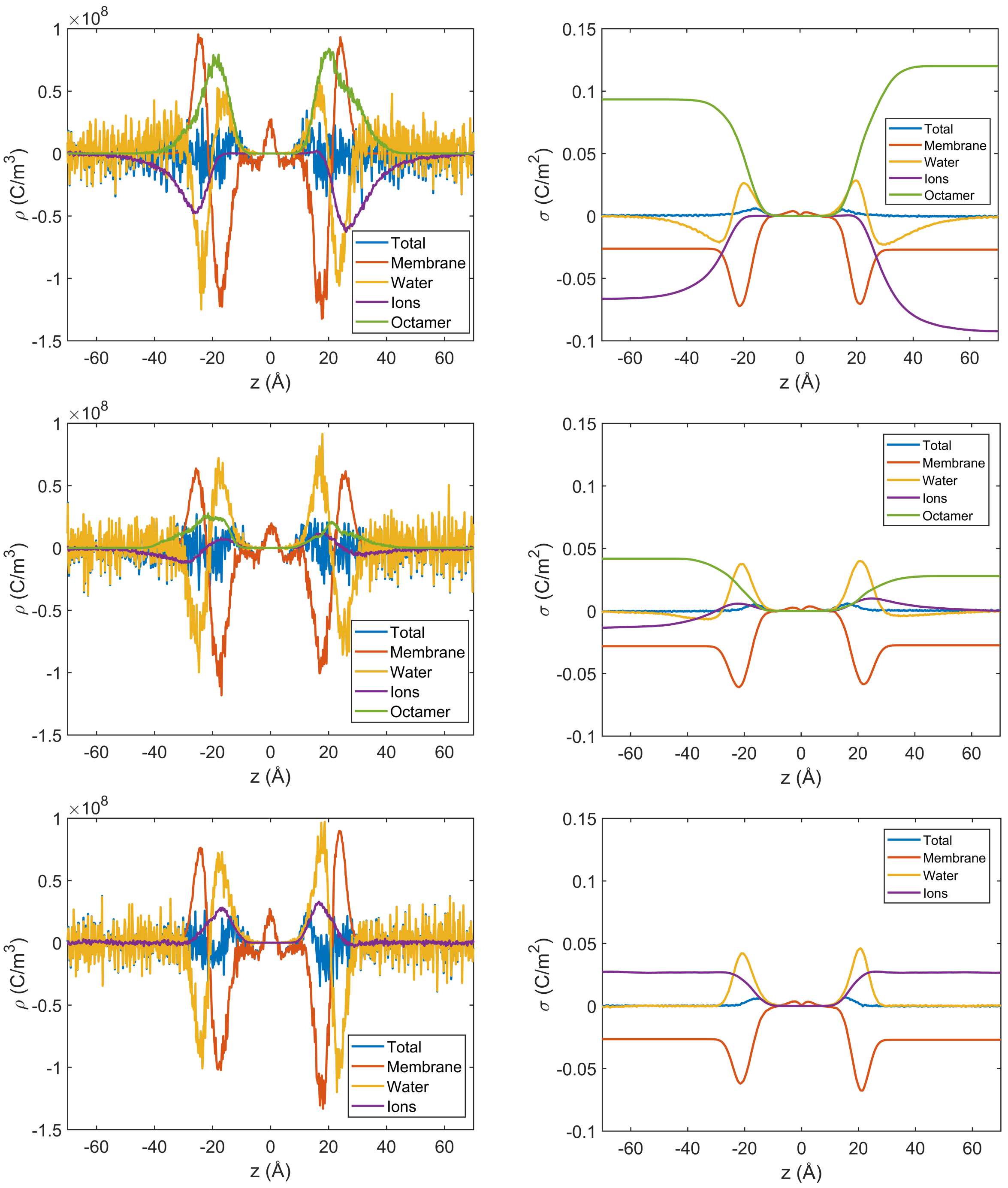

Figure S4. Charge density (total and from different components), $\rho(z)$, and integrated charge density (total and from different components), $\sigma(z)$, as a function of z from MD simulations (computed based on Eqs. 11-12) for Arg_8 – 9:1 DMPC/DMPG, Lys_8 – 9:1 DMPC/DMPG and 9:1 DMPC/DMPG systems. The charge distributions are averaged over 2500 frames in 50 ns production run.

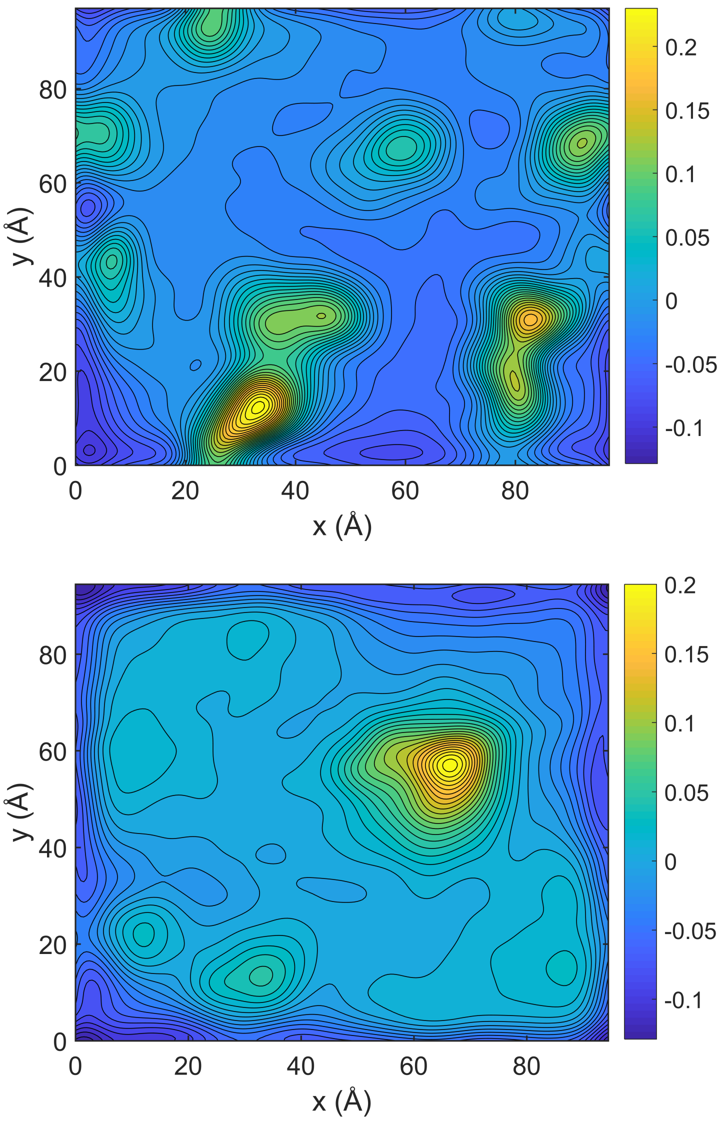

Figure S5. Electrostatic potential (in Volt) in the plane of $z = 30$ Å computed using 5000 snapshots from MD trajectories. The top panel is for the Arg_8 – 9:1 DMPC/DMPG system, and the bottom is for the Lys_8 – 9:1 DMPC/DMPG system. There is significant level of heterogeneity in the electrostatic potential due to the heterogeneous adsorption of the peptides on the membrane surface.

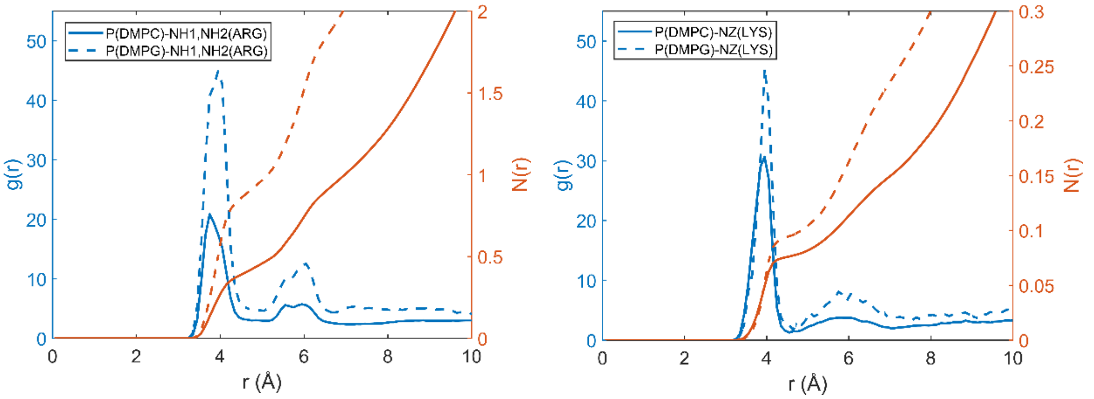

Figure S6: Radial distribution functions (RDFs) and integrated RDFs for representative atoms in the amino acid side chains (left: Arg NH1/NH2; right: Lys NZ) with respect to DMPC/DMPG phosphorus atoms from atomistic simulations of $10 \times 10 \times 18$ nm^3 systems. Each RDF is averaged over 1000 frames (~50 ns). The results highlight that the cationic residues preferentially interact with anionic lipids (DMPG).

Table S1. Acoustic and optical surface mass densities of Lys_8 and Arg_8 at maximum attachment to 9:1 DMPC/DMPG bilayers and after 10 min rinsing with polymer-free solution.[a]

	**maximum adsorption**			**after 10 min rinse**		
	**surface mass density (ng·cm^{-2})**		**θ_w, water content (%)**	**surface mass density (ng·cm^{-2})**		**θ_w, water content (%)**
	**$\Gamma_{acoustic}$**	**$\Gamma_{optical}$**		**$\Gamma_{acoustic}$**	**$\Gamma_{optical}$**	
Lys_8	34 ± 10	5 ± 3	83 ± 11	31 ± 11	5 ± 3	82 ± 12
Arg_8	210 ± 56	13 ± 2	93 ± 3.2	74 ± 19	0.8 ± 0.3	99 ± 0.8

[a] Attachment experiments were conducted in 0.1 M NaCl buffered to pH 7.4 with 0.01 M Tris at 23°C. Concentrations of Lys_8 and Arg_8 were 0.025 M. Surface mass densities were averaged over 1 min occurring at a 20 s prior to the change in solution and calculated accounting for instrumental baseline drift. Acoustic mass densities were calculated using Kelvin-Voight viscoelastic modeling for the 7th, 9th, and 11th harmonics. The water content was calculated as $\theta_w = 1 - (\Gamma_{optical} / \Gamma_{acoustic})$. Values are means ± standard deviations of at least triplicate experiments.

Table S2. Comparison of fitting parameters and free energies for Lys_8/Arg_8 and PLL/PLR

	**Arg_8**		**PLR**		**Lys_8**		**PLL**	
	Arg_8 conc. [mol. polymer/L]	Gdm conc. [mol. charge/L]	PLR conc. [mol. polymer/L]	Gdm conc. [mol. charge/L]	Lys_8 conc. [mol. polymer/L]	NH_3^+ conc. [mol. charge/L]	PLL conc. [mol. polymer/L]	NH_3^+ conc. [mol. charge/L]
**A**	0.87 ± 0.01	0.87 ± 0.01	0.88 ± 0.01	0.88 ± 0.01	0.94 ± 0.02	0.94 ± 0.01	0.93 ± 0.02	0.93 ± 0.02
**B**	-1.4 ± 0.4	-1.3 ± 0.13	-1.1 ± 0.08	-1.1 ± 0.08	-0.65 ± 0.2	-0.68 ± 0.07	-0.68 ± 0.2	-0.69 ± 0.2
K **[$\times 10^6$ M^{-1}]**	1.6 ± 0.5	0.20 ± 0 .06	44 ± 4.9	0.94 ± 0.1	1.2± 0.4	0.14 ± .04	15 ± 13	0.34 ± 0.3
n	0.54 ± 0 .09	0.54 ± 0 .09	–	–	0.52 ± 0.1	0.51 ± 0.09	–	–
**σ [C/m^2]**	0.10 ± 0.02	0.10 ± 0.01	0.17 ± 0.02	0.17 ± 0.02	0.11 ± 0.02	0.11 ± 0.01	0.28 ± 0.22	0.27 ± 0.22
**ΔG [kJ/mol]**	-45.3 ± 0.7	-40.2 ± 0.7	-54 ± 0.3	-44 ± 0.3	-44.6± 0.5	-39.3 ± 0.7	-51 ± 2	-41 ± 2
**ΔG [kcal/mol]**	-10.8	-9.6	-12.96	-10.56	-10.8	-9.36	-12.24	-9.84

Gdm is the guanidinium functional group. Charge concentrations are based on the average molecular weight of the polymer. Each repeat unit is assigned a charge of + 1.PLL and PLR data is based on fit with Gouy-Chapman Langmuir fit. For Lys and Arg, the Hill/Gouy-Chapman fit was used. A and B are fitting parameters related to the second- and third- order nonlinear susceptibilities, *K* is the apparent equilibrium constant, *n* is the Hill coefficient which describes cooperativity, and σ is the charge density.
